# The origin of sulfur in Canary Island magmas and its implications for Earth’s deep sulfur cycle

**DOI:** 10.1073/pnas.2416070122

**Published:** 2025-03-19

**Authors:** Zoltán Taracsák, Margaret E. Hartley, Ray Burgess, Marie Edmonds, Marc-Antoine Longpré, Brian D. Monteleone, Romain Tartèse, Alexandra V. Turchyn

**Affiliations:** ^a^Department of Earth Sciences, University of Cambridge, Cambridge CB2 3EQ, United Kingdom; ^b^Department of Earth and Environmental Sciences, The University of Manchester, Manchester M13 9PL, United Kingdom; ^c^School of Earth and Environmental Sciences, Queens College, City University of New York, Flushing, NY 11367; ^d^The Graduate Center, City University of New York, New York, NY 10016; ^e^Department of Geology and Geophysics, Woods Hole Oceanographic Institution, Woods Hole, MA 02543

**Keywords:** sulfur isotopes, melt inclusions, ocean island basalts, sulfur cycle

## Abstract

The global sulfur cycle plays a critical role in the redox evolution of Earth’s surface and upper mantle, yet the distribution and origin of sulfur in the mantle remains largely unconstrained. El Hierro is a volcanic island in the Canary archipelago that is fed by sulfur-rich magmas. To constrain the origin of sulfur in these melts, we combine in situ sulfur isotope analyses with regression modeling. We calculate that undegassed El Hierro melts have *δ*^34^S values of 0 ± 2‰. The average *δ*^34^S of undegassed El Hierro melts is 0.3‰ to 1‰ higher than magmas erupting at mid-ocean ridges. Mass balance calculations reveal that El Hierro’s mantle source contains 310 ± 120 μg/g sulfur and that on average 60% of sulfur in the source is of recycled origin. This recycled material should contain >1,800 μg/g sulfur to satisfy isotopic constraints on its mass fraction in the mantle source. The sulfur and oxygen isotopic signature in serpentinites and sediments deviate significantly from the upper mantle, making them unsuitable candidates for the recycled material. An oxidized partial melt of recycled oceanic crust that retained one third of its sulfur budget after subduction zone processing can explain excess sulfur in the Canary Island mantle. Recycled oceanic crust is expected to contain sulfur as sulfide, which is not capable of oxidizing the mantle. The presence of ferric iron in the recycled component is necessary to produce metasomatic melts that are oxidizing enough to carry sufficient sulfur into the mantle source of ocean island basalts.

## Significance

The sulfur isotopic composition and sulfur enrichment of magmas from the Canary Islands cannot be explained by a solely mantle origin. We suggest sulfur stored in oceanic crust that was once located near Earth’s surface is now present >90 km under the Canary Islands. Addition of an oxidized melt, originating from this recycled oceanic crust, into the mantle under the Canary Islands is needed to explain sulfur enrichment. While dissolved S^6+^ acts as an oxidizing agent in the mantle, Fe^3+^ present in the recycled crust is the primary cause of oxidation in the mantle under some ocean islands.

Silicate melts are the primary pathway through which sulfur from the Earth’s mantle can be relocated into the crust, where it plays a critical role during ore formation (1), and into the atmosphere, where it may be a constituent of aerosols cooling the Earth’s surface (2). Due to its variable oxidation state on the surface (S^2-^ to S^6+^), sulfur is an important part of Earth’s redox cycle (3). The S contents of silicate melts are highly variable (<1,000 to >6,000 μg/g) among different tectonic settings: Magmas formed at volcanic arcs are enriched in S compared to mid-ocean ridge basalts (MORBs) (4, 5), while intraplate magmas often have intermediate S contents between MORBs and arc magmas (6, 7). However, some intraplate magmas, such as those feeding the island of El Hierro (Canary Islands) (8, 9), are unusually sulfur-rich. Sulfur contents in MORBs are consistent with the melting of a depleted mantle source (DMM) containing 100 to 200 μg/g S (10). The high S content of arc magmas likely reflects transfer of S from the subducting slab into the mantle wedge (4, 11–13). On the other hand, The origin of elevated S contents in some intraplate magmas remains enigmatic, obscuring a large segment of Earth’s deep sulfur cycle.

Sulfur isotopes, conventionally expressed as *δ*^34^S in a permil notation, corresponding to the deviation of the ^34^S/^32^S ratio to that of the Vienna Canyon Diablo Troilite standard, are powerful tracers of the origin of sulfur in magmatic systems (11–14). There exists a large range in *δ*^34^S on Earth’s surface relative to deeper reservoirs. Surface lithologies contain both microbially mediated sulfide minerals that can reach very low *δ*^34^S values of <−30‰ and seawater-derived sulfur that may reach +30‰ (e.g. ref. 15). By contrast, Earth’s mantle and core have a restricted *δ*^34^S range between −1 and 0‰ (16).

**Fig. 1.**
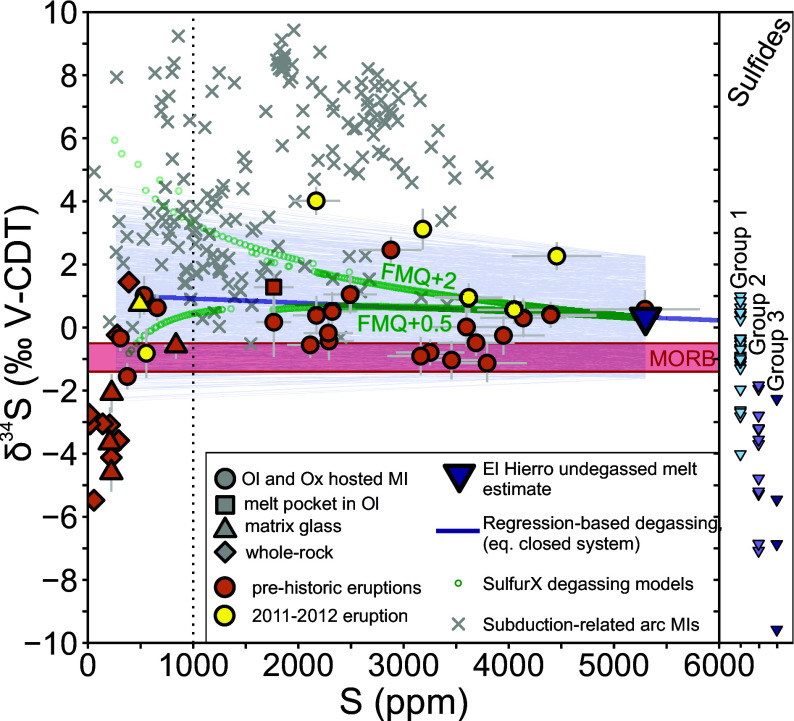
Sulfur isotope ratio vs. S content of El Hierro samples. Orange and yellow symbols are data for prehistoric and the 2011–2012 eruptions, respectively. Literature data from volcanic arc glasses (gray crosses) are from refs. 11, 14, and 13. The red band indicates the *δ*^34^S composition of DMM (18). On the *Right* side of the figure, El Hierro sulfide *δ*^34^S values measured by Beaudry et al. (22) are plotted, using their classification. The dark blue solid line is a regression fitted to MI and glass data containing >500 μg/g S assuming closed system degassing. The dark blue triangle shows the modeled undegassed melt composition. Green (for sulfide undersaturated melt) and magenta (for sulfide saturated melt) circles are results of forward degassing models calculated using SulfurX (29) at an oxygen fugacity of FMQ+0.5 and FMQ+2 (see *SI Appendix* for detail). Light blue lines are 1,000 degassing pathways calculated by randomly sampling the one SE intervals of the modeled gas-melt fractionation factor (0.9993 ± 0.0016) and the undegassed melt *δ*^34^S (+0.3 ± 1.9‰) values. Error bars are 1*σ* and defined following the protocols described in ref. 13.

Volcanic gases, whole-rock samples, and melt inclusions (MIs) from volcanic arcs all exhibit positive *δ*^34^S between +1 and +9‰ (11–14, 17), whereas glassy MORB samples have *δ*^34^S near −1‰ (16, 18) (Fig. 1). This points to a process that preferentially transfers ^34^S from subducting slabs to the mantle wedge on a global scale (11–13). Comparatively few *δ*^34^S data exist from intraplate settings. Largely undegassed Icelandic subglacial glasses from on-rift and off-rift settings have *δ*^34^S between −2.5 to −0.1‰ (19). Individual sulfide grains from the Cook, Pitcairn, and Canary Islands (20–22) have *δ*^34^S varying from −10 to +1‰, while submarine glass data from the Pitcairn and Samoan Islands fall between −1 and +3‰ (23–25). Subaerial lavas from the Cook Islands and Samoa have *δ*^34^S between −5 and +4‰ (24, 26). However, submarine glass, subaerial lava, and sulfide data available from OIBs may all be affected by degassing-driven sulfur isotope fractionation. Isotope fractionation caused by degassing may be identified and corrected for using in situ *δ*^34^S analyses of MIs with variable S contents, which represent the degassing pathway of the melt (13). For mantle-derived magmas, only a few data points, including sulfide grains, highly degassed lava samples, and Icelandic subglacial glasses, display ^32^S enrichment (19–22, 26), despite the fact that preferential removal of ^34^S during subduction (11–13) should lead to the formation of ^32^S-enriched recycled lithologies in the deeper mantle over geological time.

Here, we present sulfur isotope measurements in MIs and matrix glasses from El Hierro (Canary Islands), alongside *δ*^34^S data from bulk lava samples (Fig. 1) to investigate the causes of sulfur enrichment in these magmas. We use samples that have been extensively studied for their volatile and trace element contents and redox state (8, 9, 27), providing a globally unique and detailed framework for the interpretation of our *δ*^34^S data. Using regression modeling we explore the complex degassing processes affecting sulfur isotope ratios measured in volcanic samples and quantify the *δ*^34^S of mantle-derived El Hierro melts. Using models of melting constrained by trace elements, including copper, alongside Monte Carlo simulations, we model the sulfur content, the recycled sulfur proportion, and the *δ*^34^S of the El Hierro mantle source. Based on our modeling results we discuss the importance of sulfur degassing and sulfide formation in ocean island settings, the origin of sulfur heterogeneity in Earth’s mantle and their wider implications.

## Results

**Fig. 2.**
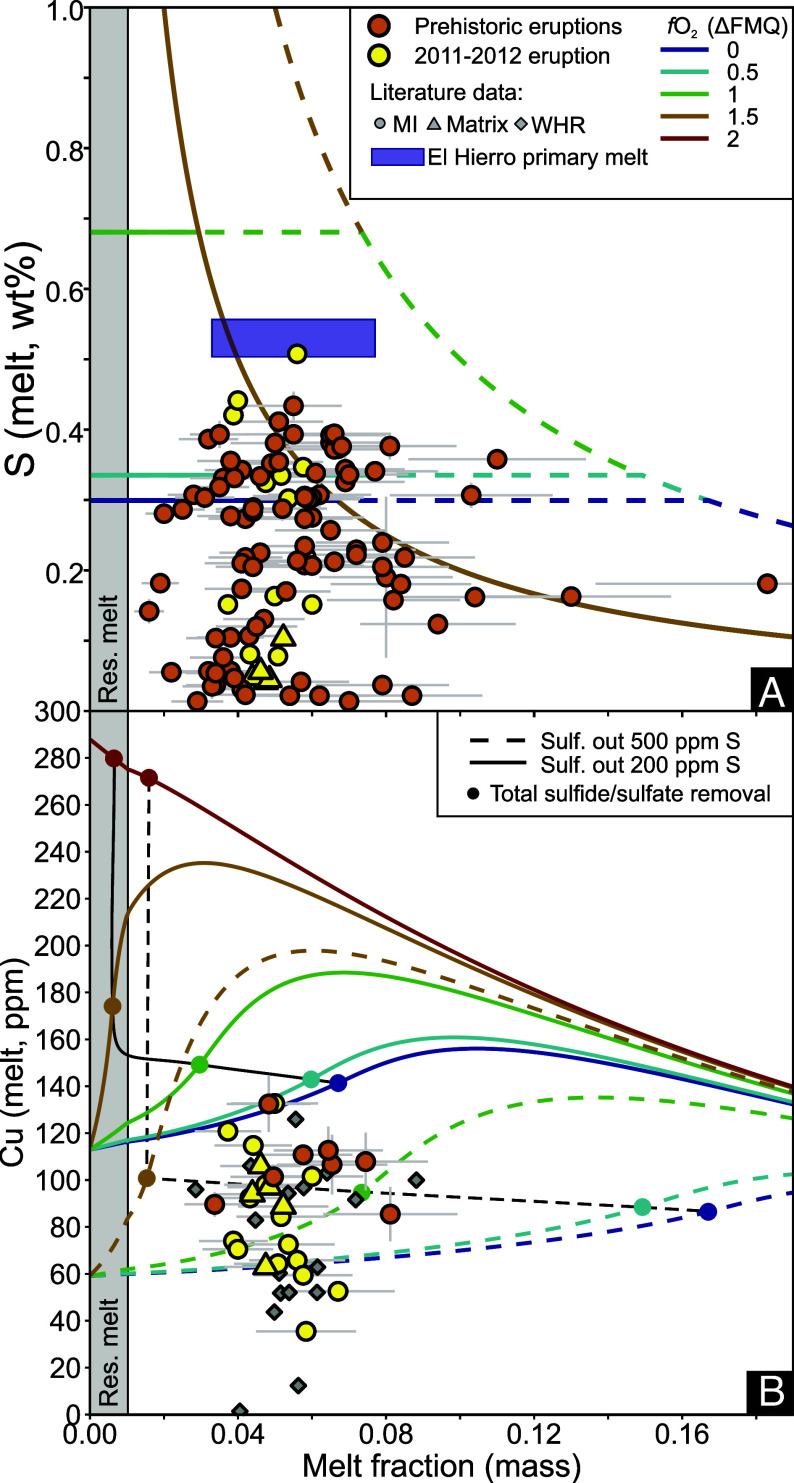
Sulfur (*A*) and copper (*B*) contents of El Hierro MIs, glasses and whole-rock samples plotted vs. melting degree. Data for glasses were originally published in refs. 8, 9, and 27. In (*A*) colored lines show the S content of mantle melts at variable *f*O_2_ (FMQ to FMQ+2) and variable mantle S content (200 μg/g—solid lines, 500 μg/g—dashed lines). Horizontal lines in (*A*) show that melt S content is buffered by sulfide or sulfate at a concentration corresponding to S solubility, while a sulfur-bearing phase is present in the solid assemblage. This is followed by perfectly incompatible behavior for S once the solid residue is exhausted in sulfur-bearing minerals—at this stage, all primary melts are sulfur undersaturated. Note that in (*A*) the red (FMQ+2) curve and the brown (FMQ+1.5) curves fully overlap. In (*B*) the same melting models are plotted for Cu. Filled circles show melting degrees upon which sulfide or sulfate is exhausted in the models. Black lines in (*B*) indicate Cu contents at which the sulfur-bearing phase becomes exhausted at 200 (solid) and 500 μg/g (dashed) mantle S content, respectively. The gray area indicates F < 0.01, during which no melt extraction occurs in the models—at these melting degrees, the composition of the residual melt is plotted. Smaller symbols in (*B*) show whole-rock data collected from El Hierro samples (41). Error bars are 1*σ*.

Sulfur isotope ratios in glasses (Fig. 1) were analyzed by secondary ion mass spectrometry (SIMS). Analyses were carried out at the NERC Ion Microprobe Facility, Edinburgh, and at NENIMF, Woods Hole Oceanographic Institution (WHOI), Woods Hole, Massachusetts. In the main text we only present data collected at WHOI, since instrumental matrix effects (28) and associated correction procedures made data reduction for the Edinburgh analyses challenging to interpret. Full details of the analytical methods and data reduction procedures are provided as *SI Appendix*. The sulfur contents of matrix glasses are between 200 and 840 μg/g, while those in MIs and embayments vary from 220 to 5,300 μg/g (Figs. 1 and 2). Whole-rock S contents are <400 μg/g (Fig. 1). Sulfur isotope ratios are between −4.6‰ to +0.7‰ for matrix glasses and between −1.6 and +4.0‰ for MIs and embayments (Fig. 1). Nine out of ten analyzed whole-rock samples have *δ*^34^S between −5.8 and +1.4‰ (Fig. 1); one whole-rock sample has a *δ*^34^S of −15.5‰.

## Discussion

### Complex Isotope Fractionation During Melt Evolution.

Sulfur isotope ratios measured in volcanic glasses and lava samples are affected by degassing (13, 17, 30). Previous models implied sulfur is degassed from the melt at relatively low pressures (<100 MPa), corresponding to the upper crust (e.g. ref. 31). This is contradicted by observations in homogenized MIs (32) and by newer degassing models (29, 33), which show that sulfur may degas at greater depth if a carbon- or water-rich fluid is present in the system. These degassing models have important implications for gas-melt sulfur equilibria and isotope fractionation during degassing, as SO_2_/H_2_S ratios in the gas are highly pressure-dependent (34).

At El Hierro, magma storage is mainly restricted to the lowermost crust and uppermost lithospheric mantle, at a depth between 15 to 20 km (27, 35, 36). The CO_2_ content of primitive El Hierro magmas is in excess of 1 wt% (9), and H_2_O content can reach 3 wt% (8, 9), hence volatile saturation and degassing must being at substantial depth in the magmatic system. This interpretation is supported by the presence of large bubbles occupying >20 vol% of MIs (9), which cannot form via shrinkage alone and therefore indicate a fluid phase was present alongside the melt upon entrapment.

Equilibrium isotope fractionation during degassing may be modeled as a fully open or closed system process. At depths of 15 to 20 km, corresponding to magma storage under El Hierro, we hypothesize that the latter process is dominant. Rapid removal of fluid bubbles is limited by the low fluid fraction of El Hierro magmas at depth, modeled at <1% by mass (9), hindering the formation of connected fluid channels that can lead to a shift from closed- to open-system degassing (37). Therefore, we assume closed-system degassing for our model calculations (Fig. 1). To investigate whether degassing can explain the relationship between *δ*^34^S and sulfur content in MIs, we fitted regressions between glass S content and *δ*^34^S following the procedures of ref. 13 (Fig. 1). In summary, this involves fitting a linear regression between S and *δ*^34^S to model closed-system equilibrium degassing. The intercept and the slope of the regression can be used to quantify the gas-melt fractionation factor *α*_(*g−m*)_ and predict the *δ*^34^S of the undegassed melt, if its initial S content is known. We use 5,300 μg/g (±5% relative error) as the undegassed S content for El Hierro, based on the highest S content measured in MIs (Fig. 1).

Assuming closed system degassing for the first degassing stage (glasses with >500 μg/g S), we calculate an average *α*_(*g−m*)_ of 0.9993 ± 0.0016 (1 SE). This value is similar to previous estimates for arc magmas, which range from 0.998 to 1.000 (13, 17, 38), and somewhat lower than the estimate of Ranta et al. (19) for more reducing Icelandic magmas (1.001). We also calculated *α*_(*g−m*)_ using a forward modeling approach (Fig. 1). We combined fractionation factors collated in ref. 39 with gas SO_2_/H_2_S and melt sulfate-sulfide ratios modeled using SulfurX (29) to calculate *δ*^34^S (see *SI Appendix* for details). Calculations predict that for redox conditions above FMQ+2 (i.e. two log units above the fayalite-magnetite-quartz buffer) appropriate for El Hierro melts based on olivine-melt V partitioning (27), both the gas and the melt are dominated by oxidized S species throughout the degassing process, resulting in *α*_(*g−m*)_ values between 0.998 to 0.999, similar to those derived from our regression model (*SI Appendix*, Fig. S6).

The relationship between *δ*^34^S and melts with S contents below 500 μg/g changes significantly, to a positive correlation. This indicates that the speciation of S in the gas and melt changed, most likely due to a change in melt oxygen fugacity (*f*O_2_). Melt reduction at El Hierro may occur via S degassing (8) or oxide fractionation (27). The latter has been suggested to decrease melt *f*O_2_ by up to 2.5 log units, and hence could drive the transition of sulfate into sulfide in the melt phase (40). At lower *f*O_2_ (<FMQ+0.5), forward models predict sulfide saturation (see *SI Appendix* spreadsheets), which could be reflected in the S content and *δ*^34^S of MIs that formed during or after melt reduction. Average *δ*^34^S in monosulfide solution from El Hierro (MMS, group 1 sulfides in Fig. 1) is −0.8 ± 1.1‰ (22), therefore sulfide fractionation would drive *δ*^34^S toward positive values. Running SulfurX models at FMQ+0.5, we find that *α*_(*g−m*)_ overturns from <1 to >1 as degassing progresses (Fig. 1), which agrees with the glass data. This change in *α*_(*g−m*)_ is driven by the conversion of H_2_S to SO_2_ in the gas as pressure decreases, causing a concurrent decrease of S^6+^/ΣS in the melt due to SO_2_ degassing (*SI Appendix*, Fig S8). Therefore, a combination of oxide fractionation-driven sulfide saturation (an interpretation supported by the low Cu content of some glasses, Fig. 2*B*) followed by sulfur degassing at a lower *f*O_2_ can also explain the measured *δ*^34^S values. At El Hierro, the complex relationship between melt *δ*^34^S and S content highlights that *α*_(*g−m*)_ can vary in response to changes in magma storage depth and *f*O_2_. Analyses of *δ*^34^S in MIs with variable S content are critical to uncover the mantle-derived sulfur isotope ratio of melts and to understand the processes that may change *δ*^34^S during melt evolution.

### The Sulfur Isotope Composition of the Undegassed Melt.

Using our equilibrium closed-system degassing model, we estimate the *δ*^34^S of undegassed El Hierro melts at 5,300 μg/g S content (Fig. 1). We predict an undegassed melt *δ*^34^S of +0.3 ± 1.9‰. This range is wider than, but also fully overlaps with, values measured from MORBs (18, 42) (−0.7 ± 0.5‰) and the upper mantle estimate of Labidi et al. (16) (−1.3 ± 0.3‰). To determine whether average MORB and undegassed El Hierro melts have a different *δ*^34^S, we performed a two-tailed unequal variances *t* test (Welsch’s *t* test) using our regression-based estimate (+0.3 ± 1.9‰), and the average *δ*^34^S of our most primitive glasses containing <1.2 wt% K_2_O (−0.2 ± 0.6‰, *SI Appendix*, Fig. S4). The average *δ*^34^S of undegassed El Hierro melts is 0.3‰ to 1.1‰ higher than the MORB average at a 95% confidence level. Our undegassed melt *δ*^34^S estimate overlaps with data collected on submarine glasses from Pacific OIB with characteristic enriched mantle (EM) isotopic compositions (23–25), including EM-I: (low ^143^Nd/^144^Nd, i.e. Pitcairn Islands) and EM-II: (high ^87^Sr/^86^Sr, i.e. Samoan Islands). El Hierro melts are ^34^S-rich compared to subglacial glasses from Iceland’s active rift zones with *δ*^34^S around -2‰, which is interpreted as recording deep recycled sulfur in the Icelandic mantle (19).

### The Abundance, Distribution, and Origin of Sulfur in the Canary Islands Mantle.

It has been proposed that Earth’s mantle has subchondritic *δ*^34^S and that most of its sulfur partitioned into the core 4.5 billion years ago (16). With respect to S concentrations, estimates for the primitive mantle (PM) are between 200 to 300 μg/g (43), while the depleted mantle is thought to contain 100 to 200 μg/g S (10). Evidence of mass-independent S isotope fractionation, a process that was common at Earth’s surface in the Archean but not since (44), identified in magmatic sulfides has been proposed to indicate that recycling of surface S into the mantle began more than 2.5 Gyr ago (21). The *δ*^34^S of basaltic glasses from Pacific OIBs (23–25) and Iceland (19) point to recycled sulfur being present in the mantle, which is either enriched (23–25) or depleted (19) in ^34^S. Despite these revelations, little is known about how recycled sulfur affects the distribution of S and redox heterogeneity in the mantle, nor about the S contents of various recycled components.

**Fig. 3.**
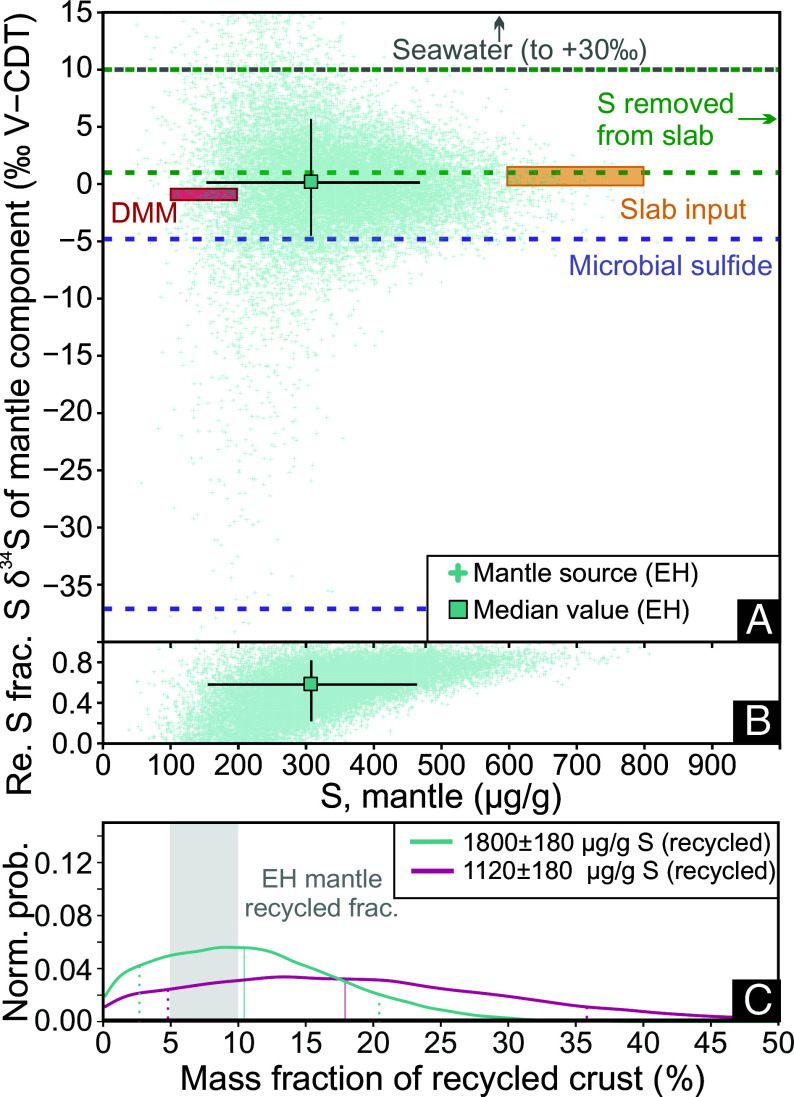
Recycled mantle component *δ*^34^S (*A*) and recycled S mass fraction (*B*) vs. mantle source S content at El Hierro, calculated using Monte Carlo simulations. Small turquoise crosses are the results of the model calculations, while the large square is the median value. The error bars in (*A* and *B*) represent values between the first and ninth decile of the sample distribution. In (*A*) Boxes and lines indicate the *δ*^34^S and S content estimates for various mantle components: slab input (orange) (12), DMM (red) (10, 18), fluids removed from the slab during subduction (green) (11–13), Phanerozoic seawater sulfate (gray, up to +30‰) (15), and microbially mediated sulfide minerals in sediments (purple lines, ODP Leg 129, Mariana basin, −4.8% to −37.1‰) (45). In (*C*), the normalized probability distribution of the recycled material mass fraction is shown; two models were calculated using 1,180 ± 180 μg/g, representing average MORB S content from ref. 5 (magenta) and 1,800 ± 180 μg/g, the minimum S content in recycled component needed to reach a 10% recycled mass fraction (turquoise). The shaded area is the proposed recycled material mass fraction present under the western Canary Islands (<10%) based on *δ*^18^O and radiogenic isotope data (41).

Using our undegassed El Hierro melt S contents and *δ*^34^S values alongside previously published lithophile and chalophile trace element data as a framework (Fig. 2) (9, 27), we model the S content of the El Hierro mantle source and the S isotopic composition of recycled material present in it (Fig. 3). We use modeling procedures detailed by Taracsák et al. (13), who applied simple mass balance to determine mantle wedge S content and slab-derived fluid S isotope composition. The model calculations require knowledge of the degree of melting alongside the S content and *δ*^34^S value of the ambient (nonrecycled) component in the mantle source.

El Hierro magmas are thought to be low-degree melts (2 to 8%) (9) of a mantle source containing <10% recycled crustal material with HIMU affinity (i.e. enriched in radiogenic Pb isotopes ^206^Pb, ^207^Pb, and ^208^Pb) (41). Incompatible trace element ratios indicate that El Hierro magmas can be generated from mantle material that has a trace element composition similar to PM (9, 41). However, due to the HIMU character of most Canary Islands magmas, a pure PM source is not realistic. Instead, we model the trace element composition of the mantle under the Western Canary Islands using three components, which together explain the trace element composition of these magmas: DMM (80%), a deep mantle-derived, high ^3^He/^4^He PM component (10%), and an eclogite partial melt from a recycled slab (10%).

To quantify melting degree (F) for El Hierro glass and whole-rock data, we use forward modeling, using the above-described three-component mantle source (see *SI Appendix* for further detail). In summary, we match modeled La/Yb ratios of mantle-derived melts calculated as a function of F with those measured in the MIs, glasses, and whole-rock samples (8, 9, 41) to determine F for each data point. These F estimates are minimally affected by crystal fractionation due to the incompatible nature of both La and Yb. The results of these calculations are presented in Fig. 2. Average melting degree calculated for El Hierro magmas is 5.5 ± 2.2% (1 *σ*).

Using the above estimate for F, the mantle source S content can be calculated assuming perfectly incompatible behavior (${\text{c}}_{\text{mantle}}$ = ${\text{c}}_{\text{melt}}$× F) if partial melting exhausted all S-bearing phases from the residual solid (Fig. 2). We explore whether S exhaustion is plausible in the El Hierro mantle source using our melting degree estimates alongside the S and Cu contents of the magmas (Fig. 2). Olivine-melt vanadium partitioning data (27), Fe-speciation measured by XANES (7), and ilmenite-magnetite equilibria (36) indicate that the *f*O_2_ of El Hierro magmas is above FMQ+1. Total S solubility (combining dissolved S^2-^ and S^6+^) at this *f*O_2_ is ∼7,000 μg/g in our model (Fig. 2), indicating that El Hierro primary melts containing ∼5,300 μg/g S (Fig. 2; see *SI Appendix* for more detail on S solubility model calculations) are sulfur-undersaturated, and therefore generated from a source that underwent total sulfide/sulfate exhaustion.

The Cu contents of El Hierro MIs are between 40 and 130 μg/g; whole-rock samples also show a similar range (Fig. 2*B*). The lower Cu contents of some El Hierro MIs and whole-rock samples likely relate to sulfide fractionation during melt evolution. Higher Cu contents, close to 130 μg/g, are more likely to be representative of the primary melt, although higher primary melt Cu contents cannot be ruled out. The behavior of Cu during mantle melting is strongly influenced by the presence or absence of sulfide (10, 46). At high *f*O_2_ (above FMQ+2) sulfide is not present in the melting assemblage (1), hence Cu behaves incompatibly, illustrated by the red line in Fig. 2*B*. Under more reducing conditions, Cu becomes more compatible with increasing sulfide fraction in the source (i.e. higher starting S content), while after sulfide exhaustion, it becomes incompatible. Total S solubility (and hence *f*O_2_) determines the rate of sulfide consumption during melting in the mantle (46), and hence the rate of change for the bulk Cu partition coefficient, controlling the shape of the curves in Fig. 2*B*. The Cu contents of El Hierro MIs are reproduced by models with 200 μg/g starting S content if *f*O_2_ is near FMQ or FMQ+0.5 (Fig. 2*B*). However, in these models, the melt S content is still close to the sulfur content at sulfide saturation, which is too low for El Hierro primary melts. Alternatively, if the mantle source contains 500 μg/g S, *f*O_2_ above FMQ+1 is required to produce melts with >90 μg/g Cu at melting degrees between 2 to 8% (Fig. 2*B*). Even if El Hierro melts are produced under more reducing conditions, only a small fraction of sulfide may be retained in the source at melt fractions upon which melt Cu contents reach >90 μg/g. Using S and Cu systematics, we predict that the solid residue becomes sulfide exhausted or retains only a minor fraction of sulfide postmelting; hence, treating S as a perfectly incompatible element in our model is realistic. If any sulfide is retained in the source, as suggested by Day et al. (41) based on highly siderophile element ratios (e.g. Pd/Os, Pt/Os) measured in El Hierro lavas, then our model provides a minimum mantle S content estimate (13).

From the modeled melting degrees, undegassed S contents, and *δ*^34^S values we calculated the S content, the fraction of recycled sulfur, and the *δ*^34^S of the recycled component within the El Hierro mantle source (Fig. 3). This model assumes the presence of two components: nonrecycled mantle, made up of an 8:1 ratio mix of DMM (150 μg/g S) and PM (250 μg/g S) with a *δ*^34^S of −0.9 ± 0.5‰, and a recycled component. To quantify uncertainties, we carried out Monte Carlo simulations. We arbitrarily define a 5% relative error for undegassed melt S content due to the lack of other contains on this uncertainty. We set the S content of the recycled component at (1,800 ± 180 μg/g), as this is the minimum concentration for S in the recycled component that satisfies previous isotopic constraints on the mass fraction (up to 10%) of recycled crustal material in the El Hierro mantle source (41).

We estimate El Hierro mantle S content at 310 ± 120 μg/g (Fig. 3*A*) and the relative proportion of recycled sulfur in the source at 58 ± 22% (Fig. 3*B*). These values indicate a significant proportion of S in the mantle originates from a source other than DMM or PM. The average and median *δ*^34^S of the recycled component is estimated at +1.7‰ and +0.1‰, with first and ninth decile values at −4.6‰ and +5.6‰.

### Implications for Earth’s Deep Sulfur Cycle and Mantle Redox Heterogeneity.

Considering that HIMU-type and oxidized magmas are common worldwide (7, 47, and references therein), recycled sulfur with a similar origin to that found under El Hierro is likely ubiquitous in the mantle. Our average and median values for the recycled component overlap with estimates of bulk subduction zone inputs in the Eastern Pacific Ocean (0 to +2‰) (12). Therefore, S in the recycled mantle component may be representative of the bulk slab that underwent minimal S isotope fractionation during subduction processing. The bulk S content of subducting slabs is estimated at <800 μg/g (12). This is too low to explain the bulk S content of the El Hierro mantle source. If the recycled component contained 800 μg/g S, then it would need to comprise >30% of the mantle melting assemblage to reproduce the observed El Hierro MI compositions. This is far in excess of the <10% threshold constrained through Pb-Os-O isotope ratios (41). After accounting for subduction processing, which likely results in S loss from the slab (11–14), the necessary mass fraction of the recycled component would only increase further.

Individual components of the subducted slab can potentially be a source of recycled S, including sediments, the oceanic crust, and serpentinites. Our estimate for the recycled component’s *δ*^34^S is near 0‰, which is similar to the upper mantle at −1‰. Therefore, any lithologies with *δ*^34^S that significantly deviate from mantle values are unlikely candidates, as they would cause a large shift in mantle *δ*^34^S due to the high fraction of recycled sulfur estimated in the El Hierro mantle source (Fig. 3*B*). Modern sediments deviate too much into positive (sulfate-rich) and negative (sulfide-rich) *δ*^34^S values (12). Paleoproterozoic sediments are much closer to mantle values at +2 to +5‰ (15); however, their high *δ*^18^O (48) rules them out, as El Hierro has mantle-like *δ*^18^O at +5.4‰ (41). Serpentines are highly heterogeneous in their sulfur contents; median S contents in high-pressure serpentinites are between 300 and 1,700 μg/g (49). The most S-rich serpentinites have positive *δ*^34^S reaching +8‰ (49), which is too high for our modeled recycled component, making serpentinites unlikely as the source of recycled sulfur. The oceanic crust can contain enough sulfur, particularly after it undergoes fractionation that increases S solubility in MORBs (5), and in some cases, its isotopic composition shifts only slightly toward positive values after alteration (12). Oxidative seafloor alteration dominantly decreases the S content of the oceanic crust (50), while mixing of hydrothermal fluids with seawater results in anhydrite and sulfide mineralization (51). Microbially meditated sulfate reduction also adds sulfide to the oceanic crust (50). Nonetheless, after subduction devolatilization, the oceanic crust is likely to be depleted in sulfur, as it is the most likely lithology that can supply sulfur to the mantle wedge with oxidized S (12, 13). Therefore, we conclude that no single lithology alone can explain the S content and *δ*^34^S of the El Hierro mantle.

If the formation of the El Hierro mantle source involves reactions with melts that originate from a recycled oceanic crust (see *SI Appendix*, Fig. S7 for detail), as we assume for our trace element-based melting degree models, it is possible to explain the high S content of the recycled component under specific conditions. We model that high-degree experimental melts (10 to 40%) of oceanic crust (52) have low sulfide solubility, between 600 to 700 μg/g. However, sulfate solubility of these melts is considerably higher at >1.5 wt% (see *SI Appendix* for detail). Therefore, by melting oceanic crust that contains as little as 300 μg/g S (i.e. up to 70% of sulfur is lost from the slab to the mantle wedge during subduction) under sufficiently oxidizing conditions that produce melts with S^6+^/ΣS above 0.6, the sulfur solubility of the melt would be high enough (>1,800 μg/g) to match the expected S content of the recycled component. In fact, the higher the S^6+^/ΣS of the melt, the lower the recycled material mass fraction required to produce a sufficiently S-rich mantle source under the Canary Islands.

If a melt of a recycled oceanic crust with relatively high S^6+^/ΣS is added to the upper mantle, it may seem that sulfur is acting as an oxidant in the upper mantle. We argue the high S^6+^/ΣS of these melts would be a consequence of ferric iron being present in the recycled crust, not sulfate. A melt with an Fe^3+^/ΣFe above 0.15 is needed to produce oceanic crust partial melts with S^6+^/ΣS above 0.6 (*SI Appendix*). We propose that as Fe^3+^ from silicates and S^2-^ sulfides dissolve into melt, equilibrium is reached by Fe^3+^ accepting electrons from S^2-^ in the melt, oxidizing it to S^6+^. Melts with high S^6+^/ΣS are then added to the upper mantle, precipitate sulfide, and oxidize silicates. During the formation of El Hierro primary melts, Fe^3+^ will again accept electrons from S^2-^, resulting in oxidizing and sulfur-rich OIB melts. This mechanism contrasts with that occurring in subduction zones, where sulfate directly present in the slab lithologies is at least partially responsible for the oxidation of arc magmas.

## Materials and Methods

We used floating rocks from the 2011–2012 eruption of El Hierro alongside prehistoric subaerial tephra and lava samples that have been extensively studied (8, 9, 27). Melt inclusions (MIs) from subaerial samples are exclusively from scoriaceous tephra deposits; lava samples were collected, where possible, from flows connected to the same scoria cones. Major elements, Cl and S, in MIs were analyzed using a Cameca SX100 electron microprobe analyzer at the University of Manchester, following procedures described by Taracsák et al. (9). The majority of electron microprobe data used here were presented (9) together with trace element data measured in the same MIs using SIMS. Copper contents of MIs were measured using laser ablation inductively coupled plasma mass spectrometry (LA-ICP-MS); all LA-ICP-MS analytical details are provided in ref. 27. Sulfur isotope ratios in MIs were measured using a Cameca IMS-1280 secondary ion mass spectrometer SIMS at the NENIMF, Woods Hole Oceanographic Institution. The instrument was run in multicollection mode using two electron multipliers. Sulfur isotope ratios in lava samples were analyzed in the Goldwin Laboratory for Paleoclimate Research, University of Cambridge, using a Flash EA coupled by continuous He flow to a Delta V mass spectrometer. Further details on the SIMS and bulk sulfur isotope analyses are given in *SI Appendix*.

## Supplementary Material

Appendix 01 (PDF)

Dataset S01 (XLSX)

## Data Availability

R scripts used to carry out monte carlo simulations and the melting models are provided on Zenodo (https://doi.org/10.5281/zenodo.14975222) (53). All study data are included in the article and/or supporting information.
