# Supplementary material for: The origin of sulfur in Canary Island magmas and its implications for Earth’s deep sulfur cycle
Source: Proc Natl Acad Sci U S A. 2025 Mar 19;122(12):e2416070122. doi: 10.1073/pnas.2416070122 (PMC11962453; doi:10.1073/pnas.2416070122)
Supplement: Supplementary file 1 — Appendix 01 (PDF) [file pnas.2416070122.sapp.pdf]

# Supplementary Information for

## The origin of sulfur in Canary Islands magmas and its implications for Earth's deep sulfur cycle

**Z. Taracsák, M. E. Hartley, R. Burgess, M. Edmonds, M-A. Longpré, B.D. Monteleone, R. Tartèse and A.V. Turchyn**

**Corresponding Author: Zoltán Taracsák.**
**E-mail: zt265@cam.ac.uk**

**This PDF file includes:**

Supplementary text
Figs. S1 to S10
References for SI reference citations

## Supporting Information Text

In the supplementary material we provide a detailed description of melt inclusion (MI) major, trace, volatile element, and sulfur isotope ratio analyses. We discuss observed discrepancies between data collected at the NERC Ion Microprobe Facility, University of Edinburgh, at the Northeast National Ion Microprobe Facility, Woods Hole Oceanographic Institute (WHOI), and the data previously published by (1). Detailed descriptions of the various model calculations used in the main text are also provided.

## Methods

**Major and trace element analyses of melt inclusions and olivine hosts.** The major and volatile element (S, Cl) contents of melt inclusions (MIs), melt embayments and matrix glasses were determined at the University of Manchester using a Cameca SX-100 electron probe microanalyzer (EPMA). Most EPMA data used here were first presented in (2).

A number of newly exposed MIs from the same set of phenocrysts used by (2) were analyzed as part of this study for sulfur isotopes. Electron probe data for these MIs are provided in the supplementary spreadsheets. Analytical procedures and uncertainties associated with glass EPMA analyses are detailed in (2, 3). Olivine hosts were analysed using the same instrument; data on olivine hosts were first published in (2).

**Correction of melt inclusion compositions for post-entrapment processes.** Olivine-hosted MIs may undergo processes post-entrapment that modify the chemical composition of the glass phase (4): these include post-entrapment crystallization and melting (PEC/PEM) of olivine along the inclusions wall. This is typically accompanied by diffusive re-equilibration of Mg-$Fe^{2+}$ between the MI and the olivine host.

To correct for the effects of diffusive re-equilibration it is necessary to derive the original Fe contents of the MIs. El Hierro whole-rock samples and matrix glasses show a strong linear correlation between ΣFeO and $SiO_2$ (5), which can ben used to estimate the original Fe content of the melt upon entrapment. Original Fe contents of El Hierro MIs were calculated using a regression fitted between El Hierro whole-rock and matrix glass ΣFeO and $SiO_2$ contents (Fig. S1). We corrected for both diffusive re-equilibration and PEC/PEM using the freely available MiMIC software (6). We used an $Fe^{3+}/Fe^{2+}$ estimate of 0.28 for the correction procedures; this value correspond to an oxygen fugacity ($fO_2$) value of two to three log units above the fayalite-magnetite-quartz buffer (FMQ) depending the model used for $Fe^{3+}/Fe^{2+}$ to $fO_2$ conversion (7–9). Uncorrected and PEC corrected data are provided in the supplementary spreadsheets.

**Whole rock sulfur isotope analyses.** Thirteen lava samples were analyzed for bulk sulfur isotope ratios ($^{34}S/^{32}S$) at the Goldwin Laboratory for Paleoclimate Research, Department of Earth Sciences, University of Cambridge. Sulfur isotope analyses were carried out using ~2 g of rock powder from each sample. Initially a mixture 6 M HCl and $CrCl_2$ solution was added to the sample powders and boiled on a hotplate for approximately 2 hours in a nitrogen atmosphere. The $H_2S$ gas produced during this process was passed through an acid trap and then bubbled in a Zn-acetate solution, precipitating the sulfur as ZnS. This step extracts reduced sulfur species ($S^{2-}$ and $S^0$) from the samples by forming $H_2S$ through reactions

$$2H^+ + S^{2-} = H_2S \qquad [1]$$

and

$$S^0 + 2Cr^{2+} + 2H^+ = H_2S + 2Cr^{3+} \qquad [2]$$

After the first step, 30 ml Thode's solution (HI + $H_2PO_3$ + HCl) was injected into the reaction vessel, and reacted with the sample for ~3 hours to extract any oxidized sulfur from the samples as $H_2S$ via the reaction:

$$SO_2{}^{4-} + 10H^+ + 8I^- = 4I_2 + H_2S + 4H_2O \qquad [3]$$

The $H_2S$ that formed during the process was passed through an acid trap and then precipitated as ZnS in a separate centrifuge tube of Zn-acetate solution. Both ZnS precipitates were then mixed with $AgNO_3$ to form $Ag_2S$, which was then dried and weighed before combustion and analysis. Sulfur isotope ratio measurements were carried out using a Flash elemental analyzer coupled by continuous helium flow Thermo Finnegan Delta V Plus gas-source isotope ratio mass spectrometer

(GS-IRMS). Blank extractions containing no sample powder were also carried out to check for any impurities in the chemical solutions used during the procedure. Blank extractions contained <0.2 ppm sulfur. Errors associated with the analyses are ±0.4‰ ($2\sigma$).

Bulk sulfur contents of lava samples were measured using colorimetry. Approximately 200 $\mu$l of sample solution containing the precipitated ZnS was reacted with HCl and dimethyl-p-phenylenediamine to redissolve the $S^{2-}$ into solution. During the reaction methyline blue dye forms from the $S^{2-}$ present in the solution, causing a blue discoloration. The absorption of the sample at 445 nm was measured using an optical spectrometer. To convert measured absorption values into concentrations, a calibration curve was acquired using $Na_2S$ solutions with known dissolved S contents before unknown analysis.

**SIMS sulfur isotope ratio analyses - NERC Edinburgh Ion Microprobe Facility.** Sulfur isotope ratios in MIs, matrix glasses and melt embayments were measured at the NERC Ion Microprobe Facility, University of Edinburgh, during a two week session in November 2019, using a Cameca IMS-1270 secondary ion mass spectrometer instrument and following analytical protocols described in (10). Analyses were carried out in multi-collector mode, simultaneously measuring $^{34}S$ on a electron multiplier and $^{32}S$ on a Faraday cup detector. We used a $Cs^+$ primary ion beam with an accelerating voltage of 10 kV and a beam current of 2-3.7 nA during analyses. The primary beam diameter was set at 10 $\mu$m. A low-energy flood electron gun was used to compensate for positive charge build up on the sample surface. Secondary ions were accelerated with 10 kV from the sample surface into the mass spectrometer. Secondary ions were passed through a 60 $\mu$m transfer lens and entrance slit, followed by a 400 $\mu$m contrast aperture and finally a 2000 $\mu$m field aperture. Mass resolution was set at 3600 MRP to avoid isobaric interferences by various molecular species ($^{32}S^-$, $^{16}O_2{}^-$, $H^{31}P^-$, $^{34}S^-$, $H^{33}S^-$, $^{16}O^{18}O^-$). Measurement were preceded by 60 s of pre-sputtering and peak centring using the dynamic transfer plates. Both isotopic species were measured for 10 seconds over 10 cycles. Eight different glasses were used as standards. Standard data and details on data processing (i.e. drift correction) are provided in (10). Counting statistical errors (internal precision) were highly variable for El Hierro samples with errors ranging from > ±2.4‰ for low S samples (<330 $\mu$g/g) to ∼ ±0.3‰ ($2\sigma$) at high (up to 4500 $\mu$g/g) S content. Errors used on the figures are the largest of three independent error estimates: internal precision (counting error), external precision (standard deviation of the primary standard), or the instrumental mass fractionation (IMF) correction error (standard error of the regression used for the IMF correction). At high S content the error of the IMF correction (∼1‰, $1\sigma$) is the largest of these three errors.

**SIMS sulfur isotope ratio analyses - Northeast National Ion Microprobe Facility, Woods Hole.** A number of glasses previously analyzed by (1) alongside a subset of glasses analyzed in 2019 in Edinburgh were reanalyzed in 2024 January at the Northeast National Ion Microprobe Facility, WHOI, using different analytical procedures. These data were collected on a IMS-1280 instrument in multi-collector mode, simultaneously measuring $^{30}Si$, $^{32}S$, $^{34}S$ on the L2, C, and H2 electron multipliers. A 210 to 260 pA $Cs^+$ primary ion beam with a diameter of 10 $\mu$m and an accelerating voltage of 10 kV was used for the analyses. A low-energy flood electron gun was used to compensate for positive charge build. Secondary ions were accelerated from the surface using a 10 kV sample voltage, passed through a 80 $\mu$m entrance slit, followed by a 400 $\mu$m contrast aperture and a 4000 $\mu$m field aperture. Mass resolution was set at 5000 MRP. Counts were collected for 16 cycles, each consisting of 5 seconds. Prior to analyses, the sample was pre-sputtered for 90 seconds, followed by centering of the primary beam using the dt-x and dt-y deflector plates. Internal precision ($2\sigma$) varied from 0.6‰ at high S contents (> 2000 $\mu$g/g) to ∼2‰ at low S contents (∼200-300 $\mu$g/g). At least two standards were analyzed alongside the unknowns in each session. Simultaneous analyses of $^{30}Si$ allowed us to calculate S contents using $^{32}S/^{30}Si$ ratios, which was quantified by fitting a regression between S content and $^{32}S/^{30}Si$ for a set of glasses with known S content.

## Instrumental mass fractionation correction procedures for SIMS sulfur isotope analyses

During every session in Edinburgh we measured at least three different glasses with known bulk $^{34}S/^{32}S$ to monitor IMF that may arise during isotope ratio analyses by SIMS (11). We find that IMF to be significant between different standards (10). Observed IMF relative to standard EGT17-01 (i.e. IMF for EGT17-01 is fixed at 0 ‰) in Edinburgh was between -12 and +1‰. Instrumental mass fractionation measured on different glass standards remained similar during every session over the two week analytical period; IMF values measured on standards are provided in (10).

At WHOI, we found IMF between the various standards to be within analytical uncertainty. During the three analytical sessions (each separated by sample exchange), we have analyzed between two to five standard glasses. These standards included five that were also used in Edinburgh, and mid-ocean ridge glass P-1326-2, used by (1) for calibration. During the three analytical sessions at WHOI, IMF values (relative to glass standard A36) were between -0.7 and +2.2‰. Combining all the standard data collected at WHOI during these sessions, the average IMF is 0.4‰ with a standard deviation of 0.7‰($1\sigma$), which is comparable to the counting statistical error of individual analyses. These results are consistent with previous data published from WHOI on different standards, collected using a mono-collection setup with two electron multipliers (12). As all the unknown glasses analyzed at WHOI have been previously measured either at the Edinburgh or UCLA SIMS facility (1), we were able to evaluate the reproducibility of the data and identify any potential biases caused by differences in analytical setup.

For the data collected in Edinburgh, we use a sulfur content-based regression to correct for the observed IMF; this regression is consistent with the observations of (13), who found IMF on the Edinburgh IMS-1270 to correlate, either logarithmically or linearly, with glass S content. We use a logarithmic fit between standard S contents (analyzed via SIMS, calculated using $^{32}$S ion yields relative to $^{16}$O) and IMF, fixed at 0‰ for primary standard EGT17-01, to estimate the IMF for the unknown samples. Using this correction procedure 84% of standard analyses can be reproduced within $2\sigma$ analytical uncertainty (10).

The IMF-corrected Edinburgh data, the data collected at WHOI, and those published by (1) all show different relationships to sulfur content (Fig. S2). IMF uncorrected data collected in Edinburgh and the data collected at UCLA by (1) both show a sharp decrease in $\delta^{34}$S with decreasing S content; this trend is amplified at low S contents. This relationship is most prominent for uncorrected Edinburgh data: melt $\delta^{34}$S decreases from $\sim +1$‰ at 3000 $\mu$g/g S content to -10‰ at 500 $\mu$g/g (Fig. S2D). Data collected at Edinburgh and corrected for IMF show the opposite relationship to S content: $\delta^{34}$S increases with deceasing S content (Fig. S2B). Once S content decreases to 1000 $\mu$g/g, this relationship overturns into a positive correlation (Fig. S2B). Data collected at WHOI show a small increase in $\delta^{34}$S with decreasing sulfur concentration up to 1000 $\mu$g/g S content, while $\delta^{34}$S decreases at S contents below that (Fig. S2A). These datasets are contradictory to each other: uncorrected Edinburgh data and the data of (1) suggest degassing under reducing conditions (see Fig S6) and an undegased melt $\delta^{34}$S that is enriched in $^{34}$S compared to the upper mantle. Corrected Edinburgh data suggest a strong preferential degassing of $^{32}$S over $^{34}$S (with a gas-melt fractionation factor $<0.997$), which is unrealistic based on our modelling (Fig S6), and an undegased melt $\delta^{34}$S that is enriched in light $^{32}$S compared to MORB.

We also directly compare data collected on the same glasses in different laboratories (Fig. S3). While a number of data points fall along the 1:1 line within 1 $\sigma$ uncertainty, a significant number of analyses do not, including most matrix glasses with low S content. As we cannot reproduce datasets collected from different laboratories, and because the results offer a way to contradictory interpretations, we only accept data collected at WHOI, which we demonstrate is not influenced by S content-dependent IMF. We hypothesize that the IMF corrected data collected in Edinburgh are still influenced by bias at S contents that are significantly different than that of the primary standard EGT17-01 (2700 $\mu$g/g). It is also likely that data collected by (1) are influenced by a similar, albeit potentially less severe, sulfur content-dependent IMF as the data we collected in Edinburgh, which was not detected due to the use of a single standard. We note that sulfide $\delta^{34}$S data of (1) are expected to be correct, as they used multiple standards to calibrate for these analyses, hence we use these data in our main text and interpretation.

## The influence of fractionation on sulfur isotope ratios

The two realistic processes that may change the S isotope ratio of the melt in a magmatic system are degassing and fractionation of sulur-bearing minerals, both of which decrease melt S content. We find considerable scatter in $\delta^{34}$S values of El Hierro glasses ($\sim \pm2$‰) at a given S content (Fig. S2A). We find that data points that strongly deviate from the $0\pm1$‰ range observed in most glasses are also the most fractionated: this is indicated by their high $K_2O$ content (Fig. S4). Least fractionated glasses remain close to the average undegassed melt $\delta^{34}$S value. Outlier analyses with positive $\delta^{34}$S include two melt inclusions hosted in spinel crystals from the 2011-2012 eruption (Fig. S2A), an olivine-hosted MI from 2011-2012 eruption and an olivine-hosted MI from a pre-historic sample. Spinels in the 2011-2012 eruption are associated with various sulfide inclusions, which have negative $\delta^{34}$S reaching -8‰ (1). Therefore, MIs hosted in spinels may have been entrapped after a molten or solid sulfate separated from the silicate melt, shifting the composition of the silicate melt towards more positive values. Other, more evolved melts that display negative $\delta^{34}$S values compared to the undegassed melt all have low S content, and are matrix glasses or degased MIs. Their formation can be explained by crystallization concurrently occurring with degassing under relatively reducing conditions (FMQ+0.5 and below). Crystallization is necessary to explain the overturn of the S-$\delta^{34}$S trends in our data, as it requires significant melt reduction via oxide fractionation (3) to occur prior to degassing (see main text for more detail). If we only use $\delta^{34}$S data collected from low $K_2O$ glasses, regardless of their S content, to estimate the primitive melt $\delta^{34}$S value, we get an average $\delta^{34}$S of $-0.2\pm0.6$‰, which is narrower than our regression-based estimates. However, the two estimates have a very similar range overall, and using either does not influence our conclusions. Nonetheless, melt fractionation may be an important factor in the observed scatter between $\delta^{34}$S and S content in El Hierro MIs and glasses.

## Modeling procedures

**Melting degree calculation for El Hierro melt inclusions.** Previous work on El Hierro whole-rock samples (14) and melt inclusions (2, 15) shows that rare earth element (REE) contents of El Hierro magmas can be modeled by 1 to 10% melting of a garnet lherzolite source lithology with average REE contents similar to that of the primitive mantle. We estimate that the lithospheric thickness under El Hierro is >120 km, using the half-space cooling model of (16) and assuming a Jurassic age for the lithosphere (17), which places the whole asthenospheric melting region in the stability field of garnet lherzolite.

By fixing mantle composition and mineralogy and using measured La/Yb values of whole-rock and glass (MI) samples, melting degree can be quantified for each analyses. We use a non-modal equilibrium melting model (Eq.4) (18) to estimate

melting degrees for El Hierro melt inclusions and whole-rock samples from which trace element data are available (2, 14, 15):

$$C_i^{melt} = \frac{C_i^{source}}{(D_i + F \times (1 - p_{bulk,i}))} \qquad [4]$$

where $C_i^{melt}$ is the concentration of element i in the melt, $C_i^{source}$ is the concentration in the source mantle, $D_i$ is the bulk distribution coefficient, $p_{i,bulk}$ is the melting proportions weighted distribution coefficient, and F is the melting degree.

We assume the melting of a garnet lherzolite lithology, and calculate phase proportions using the phase equilibria data presented by (19):

$$0.57olivine + 0.145clinopyroxene + 0.12orthopyroxene + 0.165garnet \qquad [5]$$

Melting proportions for garnet lherzolite are taken from (20):

$$0.1olivine + 0.3clinopyroxene + 0.18orthopyroxene + 0.42garnet = 1melt \qquad [6]$$

Mantle source La and Yb contents are fixed at 0.537 and 0.399 ppm, respectively. These values are calculated by mixing three mantle components in the following proportions: 10% primitive mantle (PM, La = 0.648 ppm, Yb = 0.441 ppm) (21), 80% depleted upper mantle (La = 0.23 ppm, Yb = 0.4 ppm) (22), and 10% silicate melt originating from a recycled oceanic crust, derived by melting the median eclogite composition taken from (23) by 15% (La = 2.85 ppm, Yb = 0.340). This mantle source has a near identical La/Y (1.47) to a pure primitive mantle source (1.45) (21). We set the bulk distribution coefficients (D) for La and Yb at 0.005±0.0003 and 1.09±0.13, respectively. Melting proportion-weighted distribution coefficients ($p_{bulk}$) are calculated at 0.0099±0.0003 for La and and 2.7±0.3 for Yb.

Equation 4 can be modified to calculate a ratio of two elements, rather than a single concentration. Equation 7 shows how to calculate La/Yb:

$$C_{La/Yb}^{melt} = \frac{C_{La}^{source}}{(D_{La} + F \times (1 - p_{bulk,La}))} / \frac{C_{Yb}^{source}}{(D_{Yb} + F \times (1 - p_{bulk,Yb}))} \qquad [7]$$

As we already fixed the values of every variable in Eq. 7 apart from F, we can rearrange the equation to express F, resulting in:

$$F = \frac{(\frac{C_{La}^{melt}}{C_{Yb}^{melt}} \times \frac{C_{Yb}^{source}}{C_{La}^{source}} \times D_{La}) - D_{Yb}}{1 - p_{bulk,Yb} - (\frac{C_{La}^{melt}}{C_{Yb}^{melt}} \times \frac{C_{Yb}^{source}}{C_{La}^{source}}) + (\frac{C_{La}^{melt}}{C_{Yb}^{melt}} \times \frac{C_{Yb}^{source}}{C_{La}^{source}} \times p_{bulk,La})} \quad [8]$$

Equation 8 allows melting degree to be quantified if source trace element contents and mineralogy are well characterized. After propagating the errors for source trace element contents (assuming 10% relative error), D, and $p_{bulk}$ values, we find F can be determined with relative uncertainties of ±20% to ±25%. By combining glass and whole-rock data from three publications (2, 14, 15) we estimate that average melting degree at El Hierro is 5.5±2.2% (n = 129). Various subsets of these data provide slightly different estimates, albeit all are within 1$\sigma$ uncertainty. Using data only from (15), relevant to the 2011-2012 eruption of El Hierro, melting degree is calculated at 5.0±0.8% (n = 20). Using only whole-rock data from (14) results in an estimate of 5.6±1.3% (n = 18). Data published by (2), i.e. the same glasses from which $\delta^{34}$S values were analyzed, can be divided into two groups, using the same approach described in the main text: low La/Yb (West rift) and high La/Yb (all other rifts and Tanganasoga volcano) samples. The low La/Yb samples have an average F of 9.0±3.2% (n = 19), while the high La/Yb samples have an average F of 4.9±1.6% (n = 72). The low La/Yb sample group have highly variable melting degree estimates - minimum and maximum values are 4.6% and 18.3%, respectively - this variability is reflected in their large uncertainties. For the Monte Carlo modeling presented in main text Fig. 3, we use the average melting degree estimate from all samples (5.5±2.2%) as input data.

**Degassing modelling and sulfur isotope fractionation: regression analyses and SulfurX.** The regression-based modeling approach we use to estimate sulfur isotope fractionation factors during degasing and the undegased $\delta^{34}$S of El Hierro primary magmas can be carried either assuming equilibrium closed system (see main text) or open system (13). Large bubble volumes observed in olivine-hosted melt inclusions in the studied samples (2) can only form if the fluid phase is not fully separated from the magma, supporting that closed system degassing was the prevalent process at the time of melt entrapment. Therefore, we carried out our regression-based modelling assuming equilibrium closed system degassing. However, we also modeled degassing using an equilibrium open system model, which predicts undegassed $\delta^{34}$S of +0.2±0.6‰; this value overlaps with the closed system model result. Gas-melt fraction factors predicted by the open system model are 0.9995±0.0005, which also overlap with our closed system estimate. We conclude that the assumptions made regarding open or closed system degassing do not influence our interpretation - in fact the closed system model calculation has considerably larger uncertainties, and therefore offers a more conservative estimate.

Alongside our regression-based model, we also carried out forward modeling of sulfur degasing using the openly available Python code SulfurX (24), which we combined with fractionation factors for $H_2S_{(gas)}$-$S^{2-}_{(melt)}$, $SO^{2-}_{4,(melt)}$-$S^{2-}_{(melt)}$, and $SO_{2,(gas)}$-$H_2S_{(gas)}$ listed in (25, and references therein) to calculate gas-melt fractionation factors. We tested variable input $H_2O$ (1 and 3 wt%), and $CO_2$ (1 and 2 wt%) contents for starting composition, and found that these do not significantly influence the speciation of sulfur in the melt or gas, and therefore have only a small influence on the calculated fractionation factors. Changing $fO_2$ causes the speciation of sulfur to change significantly in both the gas and melt, and therefore it exerts a primary control on fractionation factors calculated (Fig. S6). We ran models using $fO_2$ between FMQ-0.5 and FMQ+2 to explore its potential effects on S isotope fractionation during degassing.

We carried out the forward degasing model calculations assuming accumulated fractional degassing. While SulfurX assumes closed system equilibrium conditions, assuming accumulated fractional degassing was necessary to avoid abrupt changes in calculated melt and gas $\delta^{34}$S values, that sometimes changed by several permil in response to minimal (<10 MPa) change in pressure. At each calculation step, we have determined the gas-melt fractionation factor using the gas and melt sulfur speciation calculated by SulfurX, from which we calculated the sulfur isotope ratio of the gas and the melt in equilibrium. The amount of sulfur predicted to be degassed at each step was then removed from the system. Gas-melt fractionation factors were recalculated at each step based on modeled sulfur speciation in the gas and melt.

The result of the forward modeling is presented in Fig. S6. We find that at $fO_2$ values of FMQ+0.5 and below, El Hierro melts would be sulfide saturated. We note that at present, SulfurX does not take sulfide saturation into account - however, whether sulfide is saturated can be calculated from the output SCSS and SCAS values. At low $fO_2$, minimal degassing is predicted, and the melt retains 3500 $\mu$g/g S even at <10 MPa pressure. For these $fO_2$ conditions, the melt S content is not controled by degassing, but by the sulfur solubility of the melt, which is at the SCSS or slightly above ( Sulfate to total S in the melt is <0.15, Fig. S6C). For the FMQ+0.5 model we find that the melt would be initially sulfide saturated, however as degassing progresses and the melt S content decreases, the melt becomes sulfide undersaturated. This is the only model in

which the gas-melt fractionation factor changes significantly during degassing, from ∼0.9985 at high pressure to 1.0015 at low pressure (Fig. S6E). Therefore, this is the only model that can predict the observed overturn in our S isotope data (Fig. 1 main text). Degassing model calculations also shows that most sulfur degassing occurs at depth below 250 MPa (Fig. S6B). However, the lowest estimate of magma storage pressure at El Hierro, based on fluid inclusion barometry, is 280-520 MPa (5). Therefore, degassing may not be the primary driver of S loss from the melt at El Hierro at depth. Sulfide fractionation offers an alternative explanation for the observed decrease in S content in some MIs (1). While we do not find a correlation between Fe, Mg, or Ti content of the MIs and S content that would indicate that MIs with lower S content were entrapped from a melt that underwent oxide and sulfide fractionation (Fig. S5), the presence of sulfides in the 2011-2012 El Hierro eruption material, alongside the results of previous fractionation models (3) that indicate significant reduction of the melt at depth, and the abundance of large oxide phenocrysts in some El Hierro ankaramites (2) point towards conditions that facilitated sulfide saturation.

**Melting of recycled oceanic crust under OIBs, and its implilcation for the S cycle and mantle redox.** In the main text we outline our preferred model for the generation of the oxidized, sulfur-rich peridotite mantle source found under El Hierro. This model involves melting of recycled oceanic crust within the upwelling mantle under the Canary Islands, followed by reactions between these melts and a lherzolitic upper asthenosphere (Fig. S7). The recycled component have to be a melt to explain the $\delta^{34}$S systematic and S content of the El Hierro mantle source considering previous isotopic constrains on the mass fraction (<10%) of the recycled material in the source (14, 26). At certain temperature and $fO_2$, silicate melts can have high S content compared to their source (Fig. S7F). In contrast, solid lithologies (oceanic crust, serpenitines, sediments) have either too different $\delta^{34}$S or too low S content, particularly once sulfur lost to the slab during subduction zone devolatization is accounted for. Previous work suggested that between 40 to 80% of the total subducting sulfur budget reaches the deep mantle (e.g. 27).

To estimate the sulfur solubility of a partial melt of eclogitic recycled oceanic crust in the upper mantle, a major element composition estimate is necessary. We utilize glass compositions from the experiments of (28), who carried out melting experiments on MORB-like eclogites at 2-3 GPa and 1250-1500 °C - the higher end of this P and T range is applicable for the El Hierro mantle source. Their glass compositions range from basaltic to andesitic. We selected three experiments from (28) based on the amount of glass present in the final assemblage (A175, 3 GPa, 1365 °C, 8.9% melt, A202, 2.5 GPa, 1325 °C, 20.2% melt, and A184, 2 GPa, 1325 °C, 48.1% melt) to calculate SCSS (29) and SCAS (30) values for a recycled oceanic crust partial melt. At 4 GPa and 1400 °C, SCSS is estimated at 660 to 890 $\mu$g/g, while SCAS is estimated at 0.9-1.4 wt% S content (see supplementary spreadsheets).

To achieve sufficient S enrichment with the addition of less than 10% recycled material to the mantle source of El Hierro, the recycled component (melt) needs to contain at least 1800 $\mu$g/g S (see main text Fig. 3C). A reduced melt containing only $S^{2-}$ cannot dissolve sufficient amounts of S (due to its low SCSS). Therefore, the presence of $S^{6+}$ in the melt is necessary to reach sufficent S contents. Using the model of (31) we predict that for the glass composition of experiment A202 (28) (which contains 20% melt, close to the value used in our trace element model, 15%) $S^{6+}/\Sigma S$ need to be above 0.6 to reach S solubility above 1800 $\mu$g/g. These conditions are met at an $fO_2$ of FMQ+1.2 (Fig. S7G). We note that the S content of the recycled oceanic crust (eclogite) can be as low as 300 $\mu$g/g; it would still produce melts that are sulfur saturated at 1800 $\mu$g/g S content after 16% melting.

We anticipate that during slab devolatilization, most of the sulfate that was added to the oceanic crust during surface alteration (32) would be relatively mobile and removed into the mantle wedge (33), resulting in deep recycling of an oceanic crust that contains primarily sulfides as the sulfur-bearing phase. Sulfides are already the stable S-bearing phase in the mantle, therefore addition of further sulfide could not introduce redox heterogeneity to the mantle, and neither can they form melts containing sulfate without another redox sensitive element, such as Fe, being present. If the recycled oceanic crust contains sufficient ferric iron, it can produce an oxidizing melt that can destabilize sulfides in the source and dissolve these into the melt, and then convert a part of the sulfur to sulfate (while the ferric iron is reduced to ferrous iron, Fig. S7B). This melt is then added to the normal upper mantle (Fig. S7C) and reacts there, forming sulfides and high $Fe^{3+}$ silicates (such as pyroxene and garnet (Fig. S7C). Using simple mass balance calculations and assuming 10% melt addition (with 1820 ppm S, $S^{6+}/\Sigma S$ of 0.64, and FeO content of 8.25 wt%) to DMM, we calculate that the El Hierro mantle source may reach $Fe^{3+}/\Sigma Fe$ values of 0.09 - a 60% increase compared to DMM - mainly due to sulfate addition. This is due to the extreme oxidative power of $S^{6+}$: each mole of $S^{6+}$ can oxidised eight moles of $Fe^{3+}$ - on a mass basis, 100 ppm $S^{6+}$ can oxidise ∼1400 ppm Fe, or 0.18 wt% FeO.

**Continuous step melting model calculations.** To interpret our primary melt sulfur contents together with trace element systematics (REE, Cu), we constructed a continuous step melting column model based on the equations presented by (34) in combination with the sulfide-buffered melting model of (35) developed to calculate melt S contents during mantle melting. Along with sulfide we also included sulfate as a possible S-bearing mineral in the mantle source to model its effect on primary melt S and chalophile (Cu) contents. Sulfate is assumed to be present in the mantle assemblage if oxygen fugacity conditions in the mantle are above FMQ+2, in accordance with the sulfide-sulfur oxide (SSO) buffer proposed by (36), which runs near-parallel with the FMQ buffer in log($fO_2$) vs. temperature space with a +2 log unit offset.

We based our model on a melting column in which melting is driven by decompression. Melting degree in relation to pressure change is outlined in Fig. S8: for each step, pressure is decreased by dP (here we use 1 MPa steps). This decrease results in dF amount of melt being generated. We used a dF/dP value of 0.12 $GPa^{-1}$ (37). Melting degree at step $n$ in the melting column is described by Eq. 9

$$F = n \times (dP \times dF/dP) \quad [9]$$

We assume continuous (or dynamic) melting for our calculation, which requires the definition of a critical melt porosity value ($\Phi$). Once its value is reached, $\Phi$ remains constant throughout the melting process. It is calculated as $F^{res}/W^{solid}$, where $F^{res}$ is the mass of residual melt not extracted after each melting step, and $W^{solid}$ is the mass of the remaining solids. If $F < \Phi$, no melt extraction occurs and melting proceeds as equilibrium (i.e. before step $n$ is reached in Fig. S8). When $F > \Phi$, melt extraction occurs similar to fractional melting. Once F is larger than $\Phi$, the amount of extracted melt (X) can be defined, based on the model of (38) as:

$$X = \frac{F - \Phi}{1 - \Phi} \quad [10]$$

As shown in Eq. 10, X becomes positive once F reaches $\Phi$, upon which melt extraction begins: this point is represented by step $n$ in Fig. S8.

To calculate the S content of the melt at each step, we first define a $fO_2$ value, then determine the S solubility at this $fO_2$ by combining the sulfide solubility (SCSS) model of (29) and the sulfate solubility (SCAS) model of (30). Sulfate and sulfide solubilities are calculated assuming a temperature of 1500°C (equal to a $T_p$ of 1440°C) (3) and a pressure of 4 GPa (equal to ~120 km depth); note that the model of (30) is not pressure- dependent. Sulfur solubility ($C_S^{melt}$) is calculated by modelling the $S^{6+}/\Sigma S$ ratio of the mantle at the defined $fO_2$ value using the model of (31), assuming a temperature of 1500°C. We do not vary S solubility within the melting column to simplify our model calculations - uncertainties associated with current SCSS and SCAS models make more complex model calculations impractical. In our model we use fixed SCSS and SCAS values of 2560 (±340) ppm and 2.5 (±0.5) wt%, respectively, based on 14 high Mg-number (>60) whole-rock compositions published by (14).

While sulfide/sulfate is present in the mantle, melt S content is buffered at the calculated sulfur solubility. Once sulfur-bearing phases are consumed by the melting process, the S content of the melt generated at each step can be simply calculated assuming perfect incompatibility (35):

$$C_S^{melt} = C_S^{source} \times F \quad [11]$$

where $C_S^{source}$ is the S content of the source. In reality, once sulfur-rich phases are exhausted in the melting assemblage, partitioning coefficients between the silicates and the melt will control the behaviour of sulfur - however, these have been determined to be orders of magnitude smaller (0.01-0.1 for clinopyroxene, <0.001 for olivine, and <0.01 for orthopyroxene) (39) compared to those for sulfate (7.65) and sulfide (117.6) used in our model, and hence will not meaningfully affect the S content of the melt.

Estimates for silicate mineral melting proportions, used to calculate $p_{bulk}$, are from (20). Mineral proportions are estimated using the mantle phase equilibria data presented by (19).

Trace element concentrations in the melting model are calculated separately from sulfur. As melting commences upon step 1 in Fig. S8, dF amount of melt is generated. During this small step, it is reasonable to assume equilibrium is retained between the melt and the solids, hence the concentration ($C_{i,1}^{melt}$, in ppm, Eq. 12) and mass ($W_{i,1}^{melt,new}$, as mass fraction, Eq. 13) of trace element $i$ in the melt upon step 1 is calculated as

$$C_{i,1}^{melt} = \frac{C_i^{source}}{D_{i,1}} \quad [12]$$

$$W_{i,1}^{melt,new} = C_{i,1}^{melt}/10^6 \times dF \quad [13]$$

where $C_i^{source}$ is the concentration of the trace element in the unmelted source and $D_{1,i}$ is the bulk distribution coefficient during melting step 1. At the first step, the concentration and the mass of trace element $i$ in the residual melt is equal to the newly produced melt, and no melt have been extracted as F is less than Φ. After the first step, the mass $W_{i,1}^{solid}$ and concentration $C_{i,1}^{solid}$ of trace element $i$ is recalculated as:

$$W_{i,1}^{solid} = W_i^{source} - W_{i,1}^{melt,new} \quad [14]$$

$$C_{i,1}^{solid} = W_{i,1}^{solid} \times 10^6 \quad [15]$$

As each dF amount of melt is generated, the bulk distribution coefficient of the solid phase changes as different phases enter the melt in different proportions, with each phase decreasing by $p \times dF$ after every melting step. Therefore the bulk partitioning coefficient has to be recalculated at each step. This is applicable for every step during the model calculation.

At step $n$ in Fig. S8 the amount of melt reaches $\Phi$, meaning a small amount of melt ($X_n$) have to be extracted. The process to calculate extracted melt composition is explained for step *n+1*: similarly to step *1*, for step *n+1* the trace element content (in $\mu$g/g, Eq. 16) and mass (Eq. 17) of the new melt can be calculated as

$$C_{i,n+1}^{melt} = \frac{C_{n+1}^{solid}}{D_{i,n+1}} \quad [16]$$

$$W_{i,n+1}^{melt,new} = C_{i,n+1}^{melt}/10^6 \times dF \quad [17]$$

The mass of the residual melt in the instance of the melt generation (before extraction occurs) can be calculated as

$$W_{i,n+1}^{melt,res1} = W_{i,n}^{melt,res} + W_{i,n+1}^{melt,new} \quad [18]$$

After the new melt is mixed with the residual melt retained in the melting column at step $n$, a small amount of melt (dX) is removed from the column. It is calculated as:

$$dX = \frac{F_{n+1} - \Phi}{1 - \Phi} - X_n \quad [19]$$

Here dX is the change in the amount of extracted melt between step $n$ and *n+1*. The mass of trace element $i$ that is present in this dX amount of melt is added to the mass present in the melt that have been already extracted during previous steps. At each step the mass of trace element $i$ in the total extracted melt calculated using the following equation:

$$W_{i,n+1}^{melt,ext} = W_{i,n}^{melt,ext} + W_{i,n+1}^{melt,res1} \times \frac{dX}{F_{n+1} - X_n} \quad [20]$$

From Eq. 20 the concentration of trace element $i$ in the extracted melt can be calculated

$$C_{i,n+1}^{melt,ext} = \frac{W_{i,n+1}^{melt,ext}}{X_{n+1}} \times 10^6 \quad [21]$$

After the melt is extracted in step $n$, the mass of trace element $i$ in the residual melt changes to $W_{i,n+1}^{melt,res2}$, and has to be recalculated from $W_{i,n+1}^{melt,res1}$ by subtracting the amount extracted:

$$W_{i,n+1}^{melt,res2} = W_{i,n+1}^{melt,res1} - W_{i,n+1}^{melt,res1} \times \frac{dX}{F_{n+1} - X_n} \quad [22]$$

From Eq. 22 concentration of trace element $i$ can be derived for the residual melt at the end of step *n+1*:

$$C_{i,n+1}^{melt,res2} = \frac{W_{i,n+1}^{melt,res2}}{F_{n+1} - \frac{F_{n+1} - \Phi}{1 - \Phi}} \times 10^6 \quad [23]$$

Using the calculated masses of the residual and the extracted melt, which are together are the total amount of melt produced in the melting column, we can calculate both the mass ($W_{i,n+1}^{solid}$) and concentration ($C_{i,n+1}^{solid}$) of trace element $i$ in the solid residue as:

$$W_{i,n+1}^{solid} = W_i^{source} - W_{i,n+1}^{melt,res2} - W_{i,n+1}^{melt,ext} \quad [24]$$

$$C_{i,n+1}^{solid} = \frac{W_{i,n+1}^{solid}}{1 - F_{n+1}} \times 10^6 \quad [25]$$

Equations 16 to 25 are applicable to all trace elements in our melting model. We note that the presence of sulfate in the melting column does not influence the behaviour of any trace element in our model, while sulfide only influences chalcophile elements, such as Cu. Results of model calculations for various trace element contents (Ba, La, Yb, Cu) are presented in Fig. S10.

**Ratio of S/Dy in mantle melts.** Sulfur content of melt inclusions is used together with Dy as it is thought these two elements behave similarly during fractionation of mid-ocean ridge basalts (40). The S/Dy measured in melt inclusions has been used to infer the S content of primary melts and their mantle sources at various tectonic settings (2, 40–42). Here we present modeling results for different mantle lithologies (garnet and spinel lherzolite, enriched and depleted mantle) at variable $fO_2$ to show how S and Dy vary during mantle melting under different melting conditions (Fig. S9). Like for garnet (Eqs. 5 and 6), phase (Eq. 27) and melting (Eq. 26) proportions are determined for spinel lherzolite as:

$$0.72 clinopyroxene + 0.3 orthopyroxene + 0.05 spinel = 1 melt + 0.07 olivine \quad [26]$$

$$0.54 olivine + 0.135 clinopyroxene + 0.27 orthopyroxene + 0.055 spinel \quad [27]$$

If sulfide or sulfate is present in the source, these phase proportions are weighted so that all phases together add up to one.

We find that S/Dy ratios of melts remain close to constant, i.e. within ±50 which is the standard deviation in the MORB dataset of (40) and (43), only during spinel peridotite melting, and only once melting degree is above 10% (Fig. S9). The large variability in S/Dy of mantle melts is primarily controlled by two factors: the $fO_2$ dependence of S solubility in melts, and the compatible behaviour of Dy in garnet relative to spinel and other silicates (Fig. S10B). This makes S/Dy an unreliable tracer of primary melt and mantle source S contents in any geodynamic setting where melting dominantly takes place at pressures above 2-3 GPa (top of the garnet stability field at 1300-1500 °C) (44) or where $fO_2$ is >0.5 log units above the FMQ buffer (Fig. S9). This means S/Dy ratios measured in melt inclusions cannot be used in most intraplate (OIBs, intracontental volcanic fields) or subduction zone settings to estimate mantle S contents reliably. The S/Dy ratio may be a useful tool to estimate mantle S content at mid-ocean ridges, or locations where high-degree melting occurs under a thin lithosphere (e.g. Iceland). For these locations, assuming a DMM mantle composition, a S content of 150 ppm (45), and an $fO_2$ near FMQ (for MORB) or FMQ+0.5 (Iceland), we find that reasonable primary melt S/Dy ratios should be between 270 to 340, with higher values representative of lower degree melts. This is somewhat higher than MORB estimates (170-280) (40, 43), but comparable to those measured in Holuhraun, Iceland (320±86) (41)).

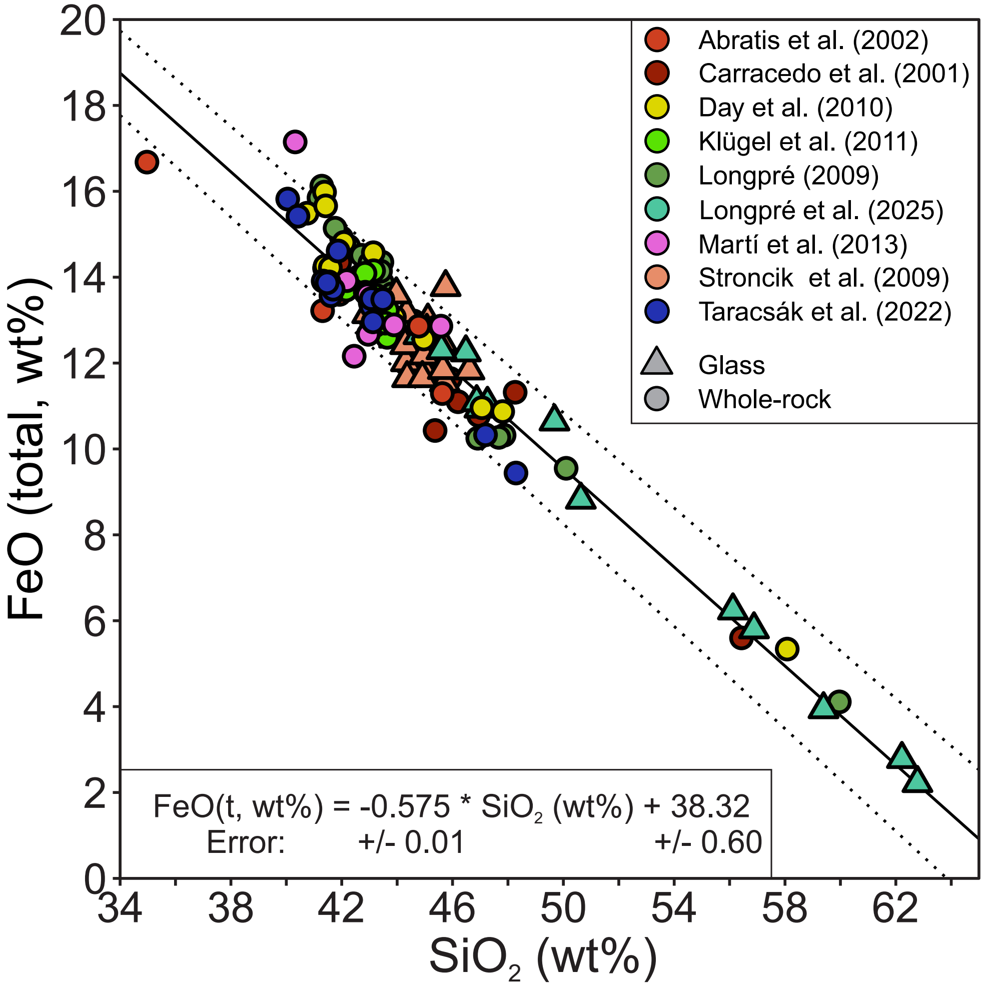


**Fig. S1.** Total FeO content versus $SiO_2$ in El Hierro glass and whole-rock samples. Data represent the 1.1 Ma subaerial activity of the island (3, 14, 46–49), and also contain a number of submarine samples (50–52). The regression line (solid black line) is calculated using simple linear regression, and is used to estimate total FeO for MIs upon entrapment. Dashed lines represent 1 standard error of the regression.

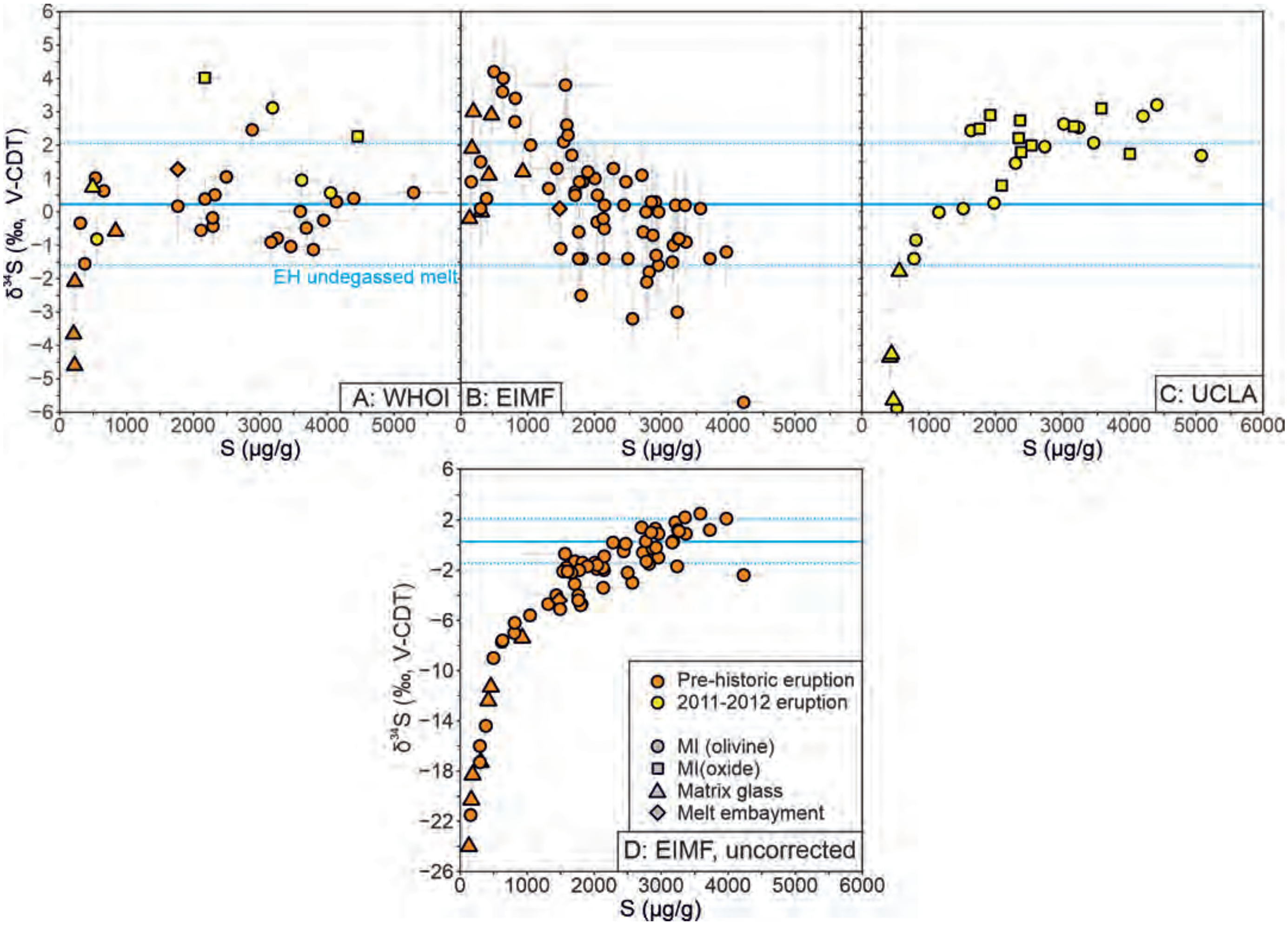


**Fig. S2.** Sulfur isotope ratio of the studied glasses, collected in different SIMS laboratories, plotted vs S content. In (A), data collected at WHOI using an EM-EM detector setup is shown. In (B), data collected at Edinburgh is plotted, after a sulfur content dependent, logarithmic-regression based IMF correction was applied to the data. In (C), data published by (1) from 2011-2012 glasses is shown. In (D), data collected in Edinburgh, uncorrected for IMF, is shown. Error bars are 1 $\sigma$. The blue solid line indicates the estimated $\delta^{34}$S of the undegased melt from Figure 1 in the main text, while blue dashed lines show the uncertainly of this estimate.

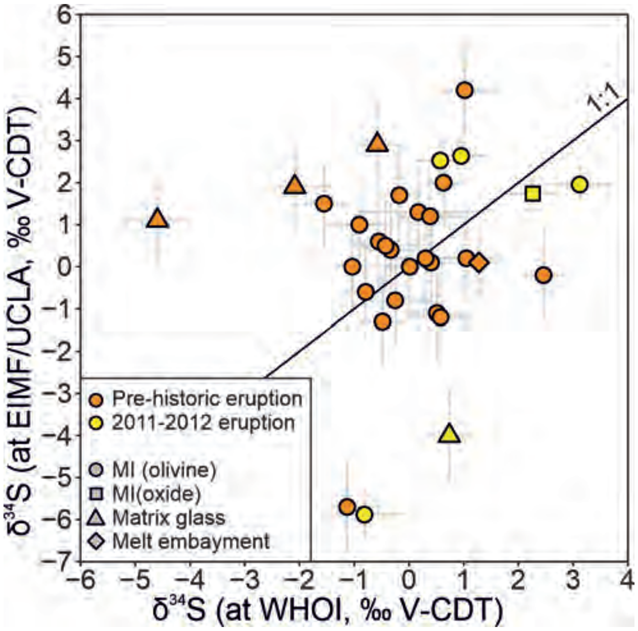


**Fig. S3.** Sulfur isotope ratio measured at Edinburgh (this work, orange symbols) or at UCLA (1) (yellow symbols) plotted against data collected at WHOI on the same glasses. Error bars are 1 $\sigma$.

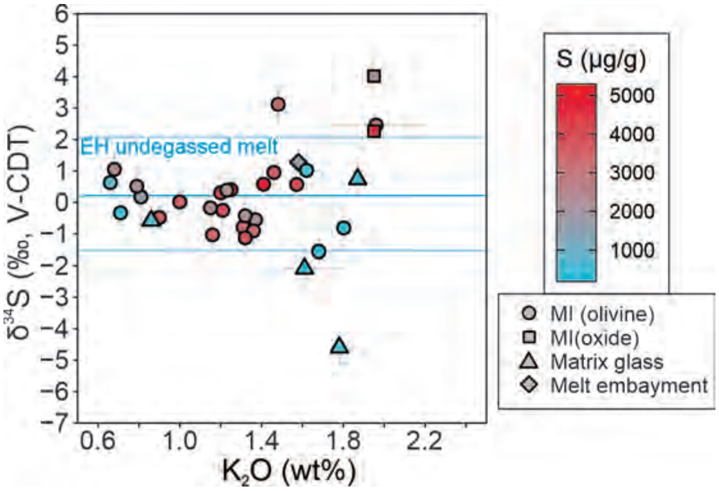


**Fig. S4.** Sulfur isotope ratio of studied glasses (showing data collected at WHOI only) vs. $K_2O$ content of the glasses. The colour of the symbols corresponds to their S content. Error bars are 1 $\sigma$.

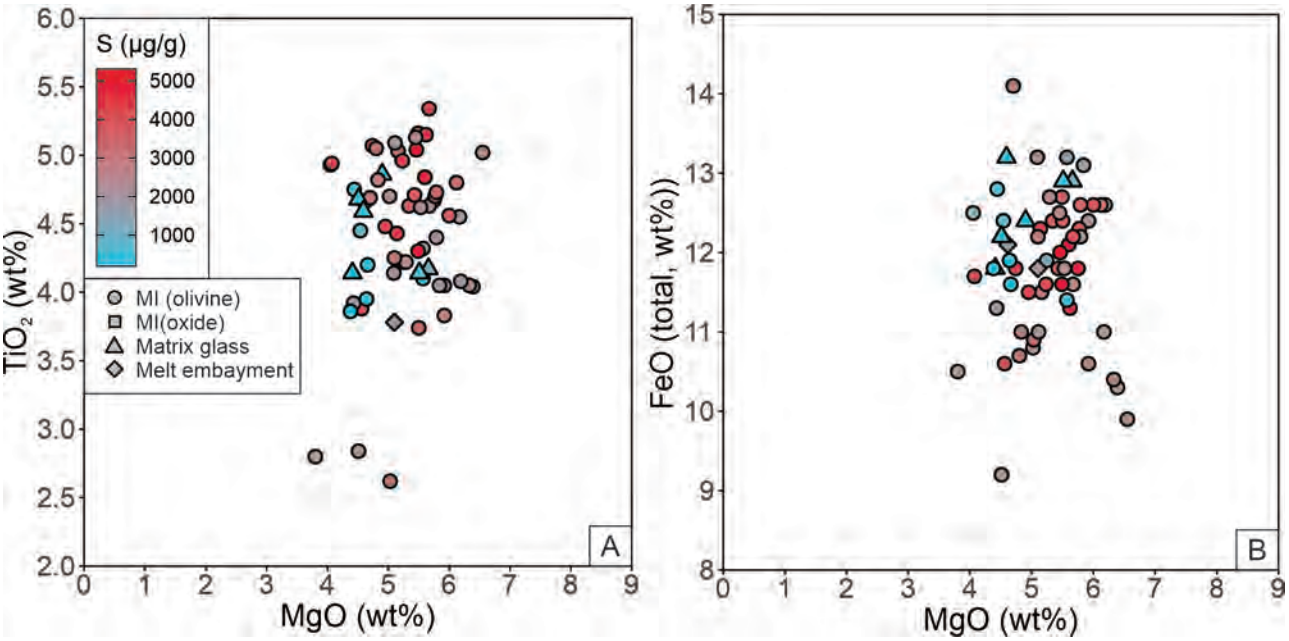


**Fig. S5.** Titanium (as $TiO_2$, A) and total FeO content (B) of studied glasses (melt inclusions data uncorrected for PEC), plotted against MgO content. Colour of the symbols uses the same scale as in Fig. S4.

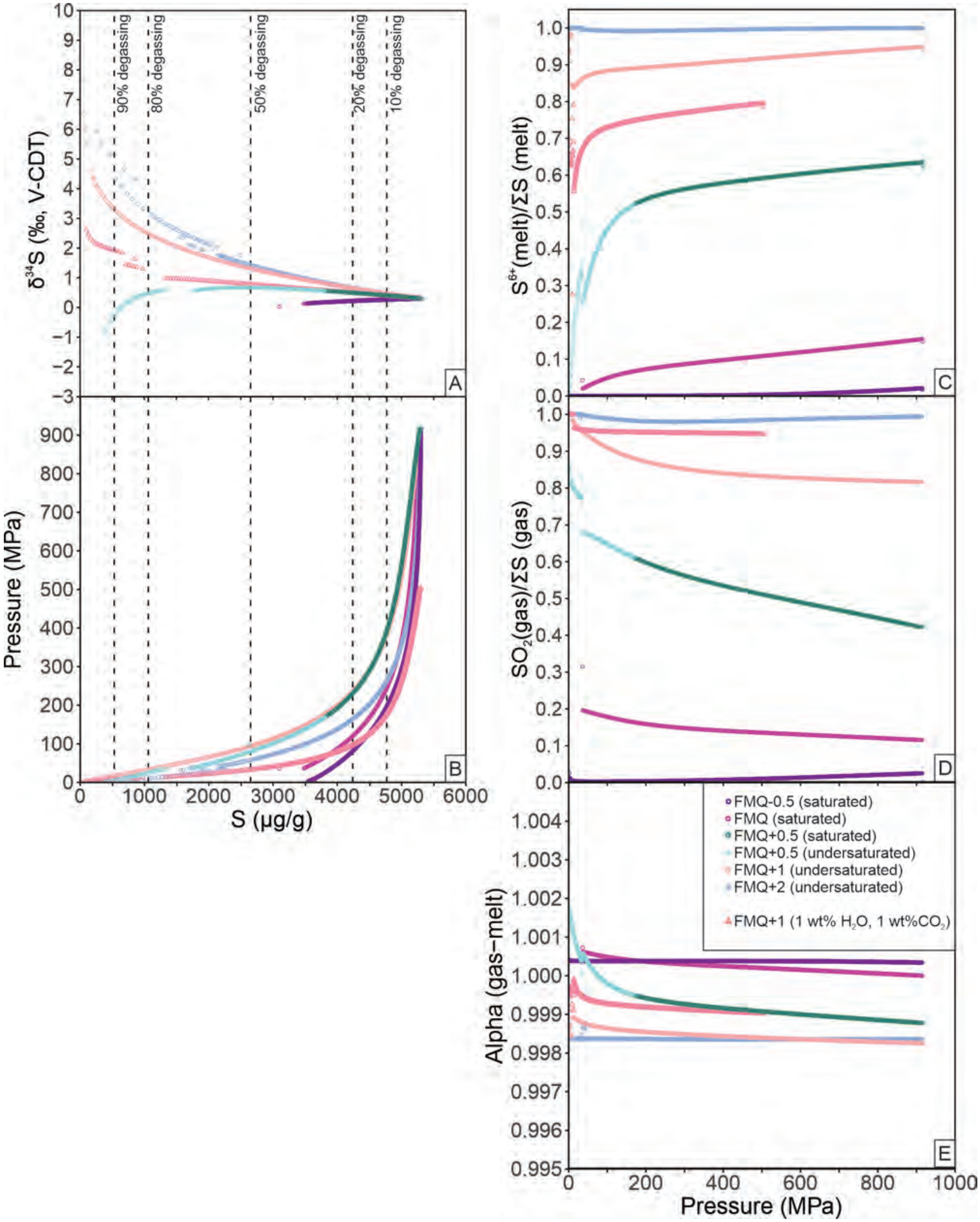


**Fig. S6.** Results of the forward degassing models calculated using SulfurX (24). Models were calculated at variable $fO_2$, from FMQ-0.5 (fully reduced sulfur in gas and melt) to FMQ+2 (fully oxidized sulfur in gas and melt). In (A), we show melt $\delta^{34}$S plotted versus S content. In (B), pressure is plotted vs. S content; dotted lines in these figures indicate the percentage (10, 20, 50, 80, and 90%) of sulfur degassing at given S content. In (C-E), melt and gas sulfur speciation, and calculated gas-melt sulfur isotope fractionation factors are plotted against pressure. Melt composition was set at the average of all analysed El Hierro melt data used in this work. For the model calculations, the water-carbon degassing model of (53), and the melt sulfur speciation model of (31) was used.

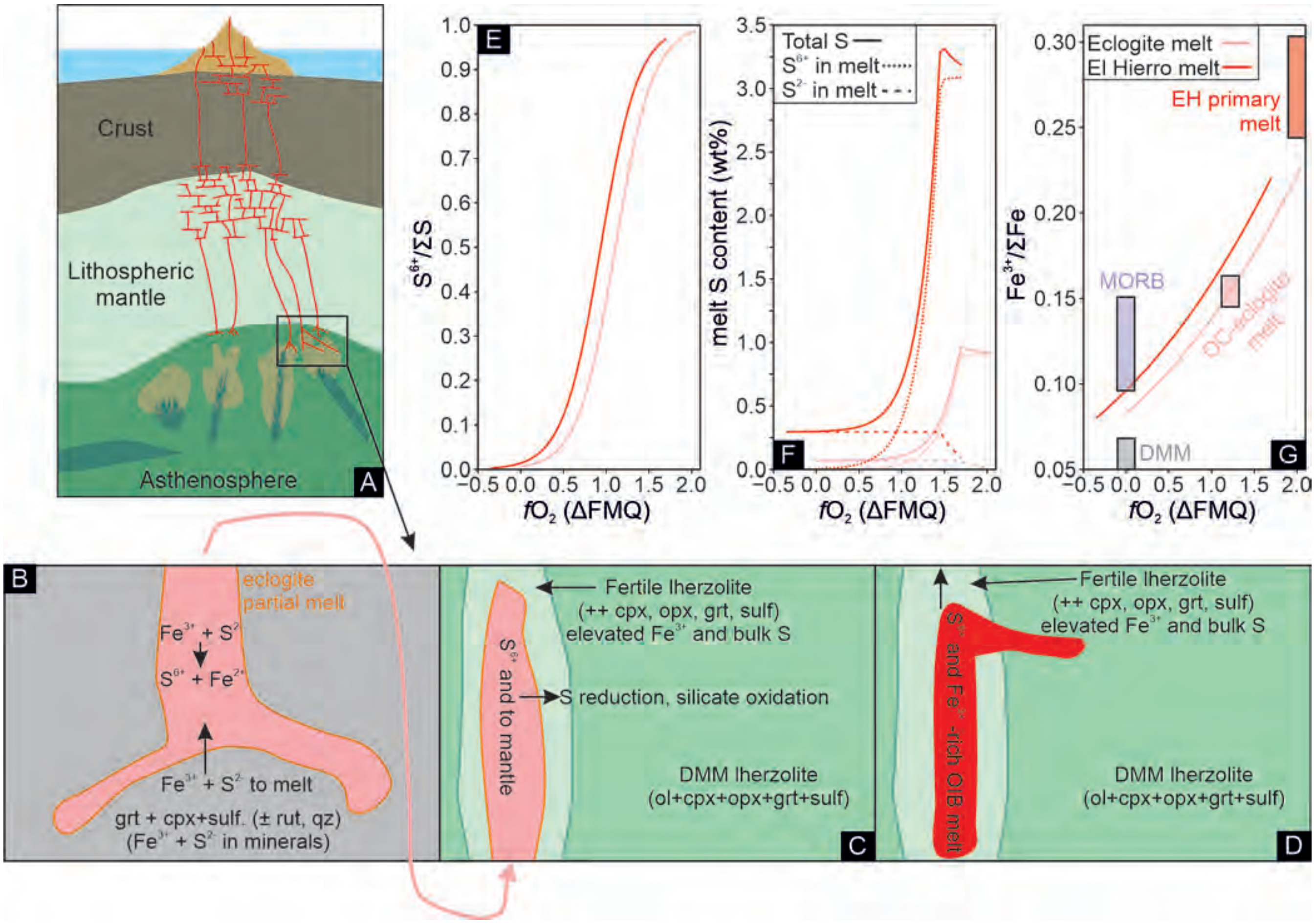


**Fig. S7.** Simple cartoon showing our preferred model for the formation of S-rich, oxidizing mantle under El Hierro (A-D), alongside melt S speciation, S content, and Fe speciation in the El Hierro primary melt and eclogite partial melt from a recycled oceanic crust (E-G). Sulfide and sulfate solubility was calculated using the model of (29) and (30), respectively. Iron speciation and $fO_2$ for MORB is from (54), while for DMM, Fe-speciation was taken from (55) ($fO_2$ for DMM was assumed to be identical to MORB). Sulfur speciation as a function of $fO_2$ was calculated using the method of (31), while Fe speciation was calculated using the method of (9).

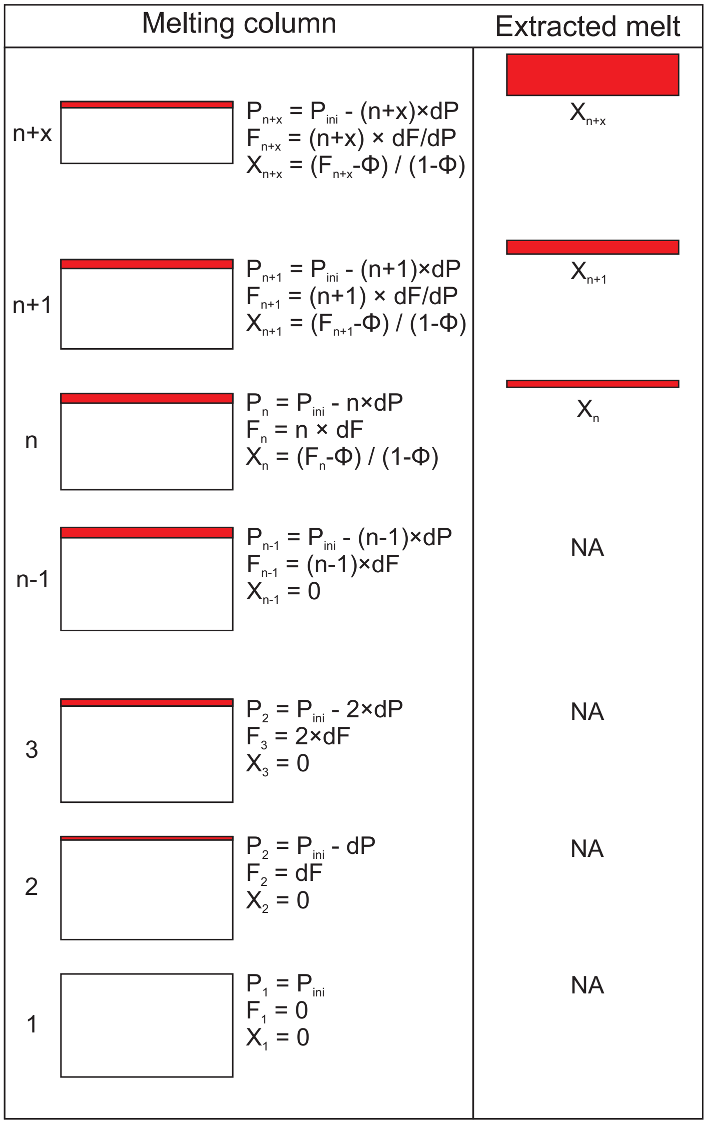


**Fig. S8.** Schematic illustration of the melting model, showing how pressure, melting degree, and the amount of the extracted melt is calculated. Note that only trace elements, including Cu, are calculated in a way where the retention of a small amount of residual melt influences the concentration in the extracted melt, while S relies on assumptions that melt S content is buffered by sulfides or sulfates at S solubility.

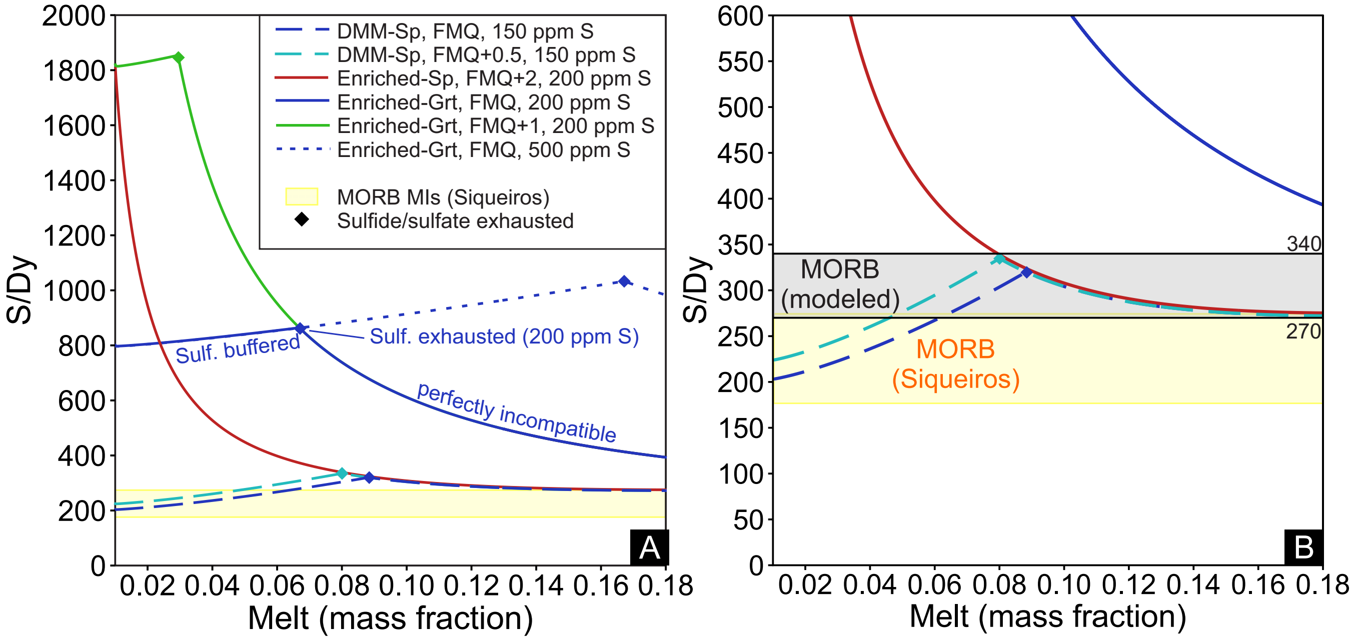


**Fig. S9.** Ratio of S and Dy in the accumulated melt versus melting degree. Various melting scenarios were plotted to demonstrate the effect of mantle S content (i.e. sulfide fraction), $fO_2$, mineral assemblage, and trace element contents (enrichment/depletion) on mantle-derived melt S/Dy ratio. Colors indicate $fO_2$ assumed for model runs. Dashed, solid, and dotted lines are models assuming 150, 200, and 500 ppm S in the mantle, respectively. Enriched mantle Dy contents are calculated as described on page 5 of the supplementary text; DMM Dy values are from (22). Diamond symbols indicate the melt fraction upon which sulfide/sulfate becomes exhausted - prior to this point melting is buffered at sulfur solubility; once passed, melting progresses assuming perfect incompatibility. Yellow area represents average S/Dy ratios measured in Siqueiros ridge MIs by (40), including 1$\sigma$ uncertainty (225$\pm$49). The larger MORB dataset of (43) has a near identical S/Dy of 236$\pm$44. The grey area in (B) indicates S/Dy ratios expected from our model calculations for MORB, which is between 270 and 340 for melting degrees above 5%.

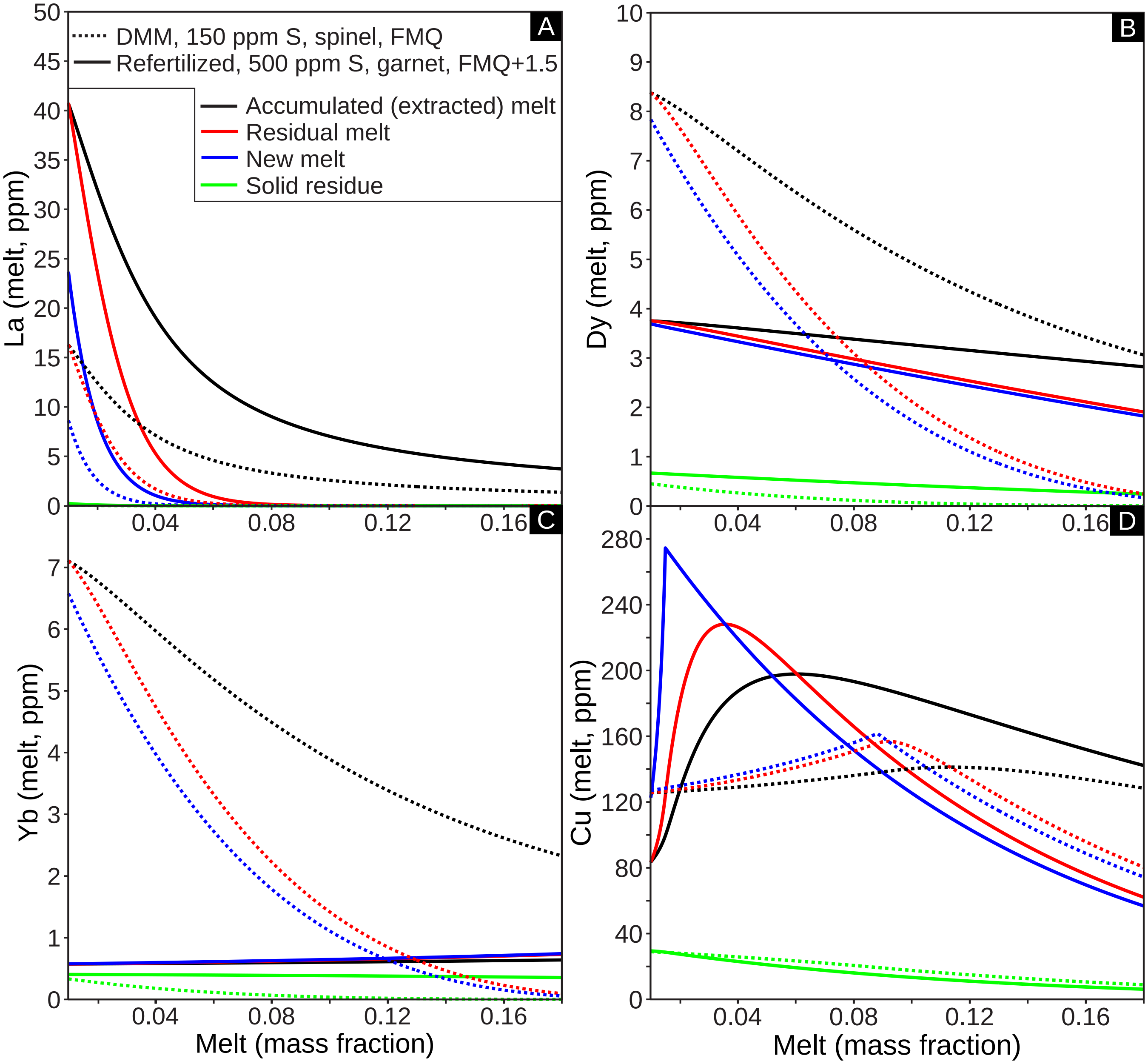


**Fig. S10.** Concentration of La (A), Dy (B), Yb (C), and Cu (D), calculated by our melting model plotted against melting degree. Green, red, blue and black lines show trace element contents in the residual solid, residual melt, the newly formed melt, and the extracted melt, respectively. Dashed lines are calculated using conditions representative for melting for MORBs, while solid lines represent our preferred model for El Hierro.

## References


1. Beaudry P, et al. (2018) Degassing-induced fractionation of multiple sulphur isotopes unveils post-Archaean recycled oceanic crust signal in hotspot lava. *Nature communications* 9(1):1–12.
2. Taracsák Z, et al. (2019) High fluxes of deep volatiles from ocean island volcanoes: Insights from El Hierro, Canary Islands. *Geochimica et Cosmochimica Acta* 258:19–36.
3. Taracsák Z, et al. (2022) Highly oxidising conditions in volatile-rich El Hierro magmas: implications for ocean island magmatism. *Journal of Petrology* 63(3):1–21.
4. Danyushevsky LV, McNeill AW, Sobolev AV (2002) Experimental and petrological studies of melt inclusions in phenocrysts from mantle-derived magmas: an overview of techniques, advantages and complications. *Chemical Geology* 183(1–4):5–24.
5. Longpré MA, Klügel A, Diehl A, Stix J (2014) Mixing in mantle magma reservoirs prior to and during the 2011–2012 eruption at El Hierro, Canary Islands. *Geology* 42(4):315–318.
6. Rasmussen DJ, Plank TA, Wallace PJ, Newcombe ME, Lowenstern JB (2020) Vapor-bubble growth in olivine-hosted melt inclusions. *American Mineralogist* 105(12):1898–1919.
7. Kress VC, Carmichael ISE (1991) The compressibility of silicate liquids containing $Fe_2O_3$ and the effect of composition, temperature, oxygen fugacity and pressure on their redox states. *Contributions to Mineralogy and Petrology* 108(1):82–92.
8. Borisov A, Behrens H, Holtz F (2018) Ferric/ferrous ratio in silicate melts: a new model for 1 atm data with special emphasis on the effects of melt composition. *Contributions to Mineralogy and Petrology* 173(12):1–15.
9. O'Neill HSC, Berry AJ, Mallmann G (2018) The oxidation state of iron in Mid-Ocean Ridge Basaltic (MORB) glasses: Implications for their petrogenesis and oxygen fugacities. *Earth and Planetary Science Letters* 504:152–162.
10. Taracsák Z, et al. (2021) Instrumental mass fractionation during sulfur isotope analysis by secondary ion mass spectrometry in natural and synthetic glasses. *Chemical Geology* 578:120318.
11. Eiler JM, Graham C, Valley JW (1997) SIMS analysis of oxygen isotopes: matrix effects in complex minerals and glasses. *Chemical Geology* 138(3-4):221–244.
12. Muth MJ, Wallace PJ (2021) Slab-derived sulfate generates oxidized basaltic magmas in the southern Cascade arc (California, USA). *Geology*.
13. Taracsák Z, et al. (2023) Sulfur from the subducted slab dominates the sulfur budget of the mantle wedge under volcanic arcs. *Earth and Planetary Science Letters* 602:117948.
14. Day JMD, Pearson DG, Macpherson CG, Lowry D, Carracedo JC (2010) Evidence for distinct proportions of subducted oceanic crust and lithosphere in HIMU-type mantle beneath El Hierro and La Palma, Canary Islands. *Geochimica et Cosmochimica Acta* 74(22):6565–6589.
15. Longpré MA, Stix J, Klügel A, Shimizu N (2017) Mantle to surface degassing of carbon- and sulphur-rich alkaline magma at El Hierro, Canary Islands. *Earth and Planetary Science Letters* 460:268–280.
16. Steinberger B, Becker TW (2018) A comparison of lithospheric thickness models. *Tectonophysics* 746:325–338.
17. Müller RD, Sdrolias M, Gaina C, Roest WR (2008) Age, spreading rates, and spreading asymmetry of the world's ocean crust. *Geochemistry, Geophysics, Geosystems* 9(4).
18. Shaw DM (1970) Trace element fractionation during anatexis. *Geochimica et Cosmochimica Acta* 34(2):237–243.
19. Stixrude L, Lithgow-Bertelloni C (2005) Thermodynamics of mantle minerals—I. Physical properties. *Geophysical Journal International* 162(2):610–632.
20. Hart SR, Gaetani GA (2006) Mantle Pb paradoxes: the sulfide solution. *Contributions to Mineralogy and Petrology* 152(3):295–308.
21. McDonough WF, Sun SS (1995) The composition of the Earth. *Chemical Geology* 120(3):223–253.
22. Salters VJM, Stracke A (2004) Composition of the depleted mantle. *Geochemistry, Geophysics, Geosystems* 5(5).
23. Barth MG, et al. (2001) Geochemistry of xenolithic eclogites from West Africa, Part I: a link between low MgO eclogites and Archean crust formation. *Geochimica et Cosmochimica Acta* 65(9):1499–1527.
24. Ding S, Plank T, Wallace PJ, Rasmussen DJ (2023) Sulfur_X: A Model of Sulfur Degassing During Magma Ascent. *Geochemistry, Geophysics, Geosystems* 24(4):e2022GC010552. e2022GC010552 2022GC010552.
25. Marini L, Moretti R, Accornero M (2011) Sulfur Isotopes in Magmatic-Hydrothermal Systems, Melts, and Magmas. *Reviews in Mineralogy and Geochemistry* 73(1):423–492.
26. Day JMD, Pearson DG, Macpherson CG, Lowry D, Carracedo JC (2009) Pyroxenite-rich mantle formed by recycled oceanic lithosphere: Oxygen-osmium isotope evidence from Canary Island lavas. *Geology* 37(6):555–558.
27. de Moor JM, Fischer TP, Plank T (2022) Constraints on the sulfur subduction cycle in Central America from sulfur isotope compositions of volcanic gases. *Chemical Geology* 588:120627.
28. Pertermann M, Hirschmann MM (2003) Anhydrous Partial Melting Experiments on MORB-like Eclogite: Phase Relations, Phase Compositions and Mineral–Melt Partitioning of Major Elements at 2-3GPa. *Journal of Petrology* 44(12):2173–2201.
29. Fortin MA, Riddle J, Desjardins-Langlais Y, Baker DR (2015) The effect of water on the sulfur concentration at sulfide

saturation (SCSS) in natural melts. *Geochimica et Cosmochimica Acta* 160:100–116.

30. Zajacz Z, Tsay A (2019) An accurate model to predict sulfur concentration at anhydrite saturation in silicate melts. *Geochimica et Cosmochimica Acta* 261:288–304.
31. O'Neill HS, Mavrogenes JA (2022) The sulfate capacities of silicate melts. *Geochimica et Cosmochimica Acta* 334:368–382.
32. Alt JC, Shanks WC (2011) Microbial sulfate reduction and the sulfur budget for a complete section of altered oceanic basalts, IODP Hole 1256D (eastern Pacific). *Earth and Planetary Science Letters* 310(1-2):73–83.
33. Walters J, Cruz-Uribe A, Marschall H (2020) Sulfur loss from subducted altered oceanic crust and implications for mantle oxidation. *Geochemical Perspectives Letters* 13:36–41.
34. Shaw DM (2000) Continous (dynamic) melting theory revisited. *The Canadian Mineralogist* 38(5):1041–1063.
35. Lee CTA, et al. (2012) Copper systematics in arc magmas and implications for crust-mantle differentiation. *Science* 336(6077):64–68.
36. Mungall JE (2002) Roasting the mantle: Slab melting and the genesis of major Au and Au-rich Cu deposits. *Geology* 30(10):915–918.
37. Langmuir CH, Klein EM, Plank T (1992) Petrological Systematics of Mid-Ocean Ridge Basalts: Constraints on Melt Generation Beneath Ocean Ridges in *Mantle Flow and Melt Generation at Mid-Ocean Ridges.* (American Geophysical Union), pp. 183–280.
38. Zou H (1998) Trace element fractionation during modal and nonmodal dynamic melting and open-system melting: a mathematical treatment. *Geochimica et Cosmochimica Acta* 62(11):1937–1945.
39. Callegaro S, et al. (2020) The quintet completed: The partitioning of sulfur between nominally volatile-free minerals and silicate melts. *American Mineralogist* 105(5):697–707.
40. Saal AE, Hauri EH, Langmuir CH, Perfit MR (2002) Vapour undersaturation in primitive mid-ocean-ridge basalt and the volatile content of Earth's upper mantle. *Nature* 419(6906):451–455.
41. Bali E, Hartley M, Halldórsson S, Gudfinnsson G, Jakobsson S (2018) Melt inclusion constraints on volatile systematics and degassing history of the 2014–2015 Holuhraun eruption, Iceland. *Contributions to Mineralogy and Petrology* 173(9).
42. Muth MJ, Wallace PJ (2022) Sulfur recycling in subduction zones and the oxygen fugacity of mafic arc magmas. *Earth and Planetary Science Letters* 599:117836.
43. Jenner FE, O'Neill HSC (2012) Analysis of 60 elements in 616 ocean floor basaltic glasses. *Geochemistry, Geophysics, Geosystems* 13(2).
44. Klemme S, O'Neill HS (2000) The near-solidus transition from garnet lherzolite to spinel lherzolite. *Contributions to Mineralogy and Petrology* 138(3):237–248.
45. Ding S, Dasgupta R (2017) The fate of sulfide during decompression melting of peridotite–implications for sulfur inventory of the morb-source depleted upper mantle. *Earth and Planetary Science Letters* 459:183–195.
46. Carracedo JC, Badiola ER, Guillou H, de la Nuez J, Torrado FP (2001) Geology and volcanology of La Palma and El Hierro, Western Canaries. *Estudios Geológicos* 57(5-6):175–273.
47. Longpré MA (2009) Phd thesis (Trinity College, University of Dublin).
48. Martí J, et al. (2013) Causes and mechanisms of the 2011–2012 El Hierro (Canary Islands) submarine eruption. *Journal of Geophysical Research: Solid Earth* 118(3):823–839.
49. Longpré MA, Klügel A, Stix J (2025) Matrix glass composition of tephra samples from El Hierro's Tanganasoga volcano and western rift (Canary Islands). Version 1.0. *EarthChem Library Dataset, Interdisciplinary Earth Data Alliance (IEDA).*
50. Abratis M, Schmincke HU, Hansteen T (2002) Composition and evolution of submarine volcanic rocks from the central and western Canary Islands. *International Journal of Earth Sciences* 91(4):562–582.
51. Klügel A, Hansteen TH, van den Bogaard P, Strauss H, Hauff F (2011) Holocene fluid venting at an extinct Cretaceous seamount, Canary archipelago. *Geology* 39(9):855–858.
52. Stroncik NA, Klügel A, Hansteen TH (2009) The magmatic plumbing system beneath El Hierro (Canary Islands): constraints from phenocrysts and naturally quenched basaltic glasses in submarine rocks. *Contributions to Mineralogy and Petrology* 157(5):593–607.
53. Newman S, Lowenstern JB (2002) VolatileCalc: a silicate melt–$H_2O$–$CO_2$ solution model written in Visual Basic for excel. *Computers & Geosciences* 28(5):597–604.
54. Berry AJ, Stewart GA, O'Neill HSC, Mallmann G, Mosselmans JFW (2018) A re-assessment of the oxidation state of iron in MORB glasses. *Earth and Planetary Science Letters* 483:114–123.
55. Davis FA, Cottrell E (2021) Partitioning of $Fe_2O_3$ in peridotite partial melting experiments over a range of oxygen fugacities elucidates ferric iron systematics in mid-ocean ridge basalts and ferric iron content of the upper mantle. *Contributions to Mineralogy and Petrology* 176(9):67.
